# The *O*-methyltransferase gene cluster in *Camptotheca acuminata* is involved in camptothecins and flavonoids metabolism

**DOI:** 10.1093/hr/uhag145

**Published:** 2026-04-21

**Authors:** Qingyan Ruan, Xiaoxuan Fan, Hao Zhan, Maohua Cai, Zengyuan Wang, Qin Zhou, Yue Feng, Yiqing Peng, Xiaolong Hao, Guoyin Kai

**Affiliations:** Laboratory of Medicinal Plant Biotechnology, School of Pharmaceutical Sciences, Zhejiang Chinese Medical University, Hangzhou 310053, China; Laboratory of Medicinal Plant Biotechnology, School of Pharmaceutical Sciences, Zhejiang Chinese Medical University, Hangzhou 310053, China; Laboratory of Medicinal Plant Biotechnology, School of Pharmaceutical Sciences, Zhejiang Chinese Medical University, Hangzhou 310053, China; Laboratory of Medicinal Plant Biotechnology, School of Pharmaceutical Sciences, Zhejiang Chinese Medical University, Hangzhou 310053, China; Laboratory of Medicinal Plant Biotechnology, School of Pharmaceutical Sciences, Zhejiang Chinese Medical University, Hangzhou 310053, China; Laboratory of Medicinal Plant Biotechnology, School of Pharmaceutical Sciences, Zhejiang Chinese Medical University, Hangzhou 310053, China; Laboratory of Medicinal Plant Biotechnology, School of Pharmaceutical Sciences, Zhejiang Chinese Medical University, Hangzhou 310053, China; Laboratory of Medicinal Plant Biotechnology, School of Pharmaceutical Sciences, Zhejiang Chinese Medical University, Hangzhou 310053, China; Laboratory of Medicinal Plant Biotechnology, School of Pharmaceutical Sciences, Zhejiang Chinese Medical University, Hangzhou 310053, China; Laboratory of Medicinal Plant Biotechnology, School of Pharmaceutical Sciences, Zhejiang Chinese Medical University, Hangzhou 310053, China

## Abstract

*Camptotheca acuminata*, a tree native to southern China and cultivated for horticultural purposes, has attracted considerable attention due to its ability to synthesize camptothecin-type compounds with potent antitumor activity. Flavonoids and camptothecins share a conserved benzene ring scaffold, suggesting potential crosstalk in their biosynthetic pathways. Metabolic profiling of 1-year-old *C. acuminata* seedlings revealed tissue-specific accumulation of *O*-methylated quercetin and camptothecin in the stem phloem. Notably, CaOMT3, one of four clustered *O*-methyltransferase genes, showed expression levels that positively correlated with rhamnetin accumulation across tissues. Transient transformation in *Nicotiana benthamiana*, coupled with heterologous expression *in vitro*, demonstrated that CaOMT1, CaOMT2, and CaOMT3 efficiently methylated 9-hydroxycamptothecin and 10-hydroxycamptothecin. Regarding their flavonoid-methylation abilities, CaOMT1 and CaOMT3 were identified as bifunctional enzymes that targeted the 4′-hydroxyl and 7-hydroxyl groups of quercetin, respectively, whereas CaOMT2 exhibited strict specificity for the 4′-hydroxyl group of quercetin. Unfortunately, CaOMT4 showed no methylation activity toward the tested substrates. The catalytic dyad and key substrate-binding sites of the CaOMTs were identified via molecular docking and subsequently verified by site-directed mutagenesis. F166, M313, and L316 were proposed to be critical for activity toward both quercetin and hydroxycamptothecin. Notably, N261, S309, and T312 were suggested as key amino acids responsible for the lack of 7-*O*-methylation activity in CaOMT2. Taken together, our findings revealed a bifunctional *OMT* gene cluster and provided molecular insights into the substrate-binding pockets.

## Introduction

*Camptotheca acuminata*, a deciduous tree endemic to China, is often regarded as an ideal landscape plant due to its tall and straight form, luxuriant foliage, and peculiar fruits. *Camptotheca acuminata* is rich in diverse indole alkaloids, which are recognized for their broad spectrum of biological activities [1–5]. Of particular note is its high content of camptothecin, a monoterpenoid indole alkaloid with potent antitumor activity, which has attracted extensive research attention [6]. Presently, irinotecan and topotecan, two derivatives of camptothecin, have been widely used in the global market for the treatment of malignant tumors [7, 8]. In addition to its capacity to synthesize a range of alkaloids, *C. acuminata* has been demonstrated to be a highly proficient producer of flavonoids [9]. A considerable number of flavonoids function as pigments and protective substances in plants [10, 11]. To date, targeted metabolomics in *C. acuminata* has revealed over 60 flavonoids and 47 alkaloids, including a significant number of methoxylated flavonoids and camptothecin [2, 9].

Further research indicates that the biosynthetic pathways for these compounds in *C. acuminata* may share common modifying enzymes [9, 11]. Both flavonoids and camptothecins are derived from aromatic amino acids via the shikimate pathway, thus sharing a similar aromatic ring structure. Focusing on camptothecin, its biosynthesis initiates with the condensation of secologanic acid and tryptamine to form the common intermediate strictosidinic acid, which then undergoes a series of reactions to ultimately yield camptothecin [12, 13]. To date, 23 enzymes involved in camptothecin biosynthesis have been functionally validated in *C. acuminata* [14–21]. Flavonoid biosynthesis, extensively studied in various species, initiates from L-phenylalanine and proceeds through enzymes including phenylalanine ammonia-lyase (PAL), cinnamate 4-hydroxylase (C4H), 4-coumarate: coenzyme A ligase (4CL), chalcone synthase (CHS), chalcone isomerase (CHI), and flavone synthase (FNS) [22, 23]. Currently, seven genes involved in this pathway have been documented in *C. acuminata* [14, 24–26]. Notably, several reported enzymes have been demonstrated to possess the capability to catalyze both types of compounds concurrently [12–15, 24]. For instance, Ca10OMT exhibits dual substrate specificity, methylating both 10-hydroxycamptothecin and flavonoids such as kaempferol and quercetin [14], further supporting the hypothesis that these pathways may share common modifying enzymes [9, 11].

*O*-methylation modifies diverse specialized metabolites, including phenylpropanoids, flavonoids, and alkaloids, thereby enhancing their chemical properties and biological activities [11, 27, 28]. Plant OMTs constitute a major enzyme family that catalyzes the SAM-dependent methylation of oxygen atoms in diverse secondary metabolites. They are classified into OMT I or OMT II based on molecular weight and divalent cation dependence [29]. The most significant difference between OMT I and OMT II is that the catalysis by OMT I is dependent on metal ions, such as Mg^2+^ and Zn^2+^, whereas OMT II catalysis does not require them [9]. Previous studies have indicated that OMT I is involved in lignin metabolism [30]. OMT II evolves later than OMT I and accommodates a broader spectrum of substrates, such as phenylpropanoids, alkaloids, and flavonoids [11, 27, 28]. To date, three CaOMTs belonging to the OMT II family have been implicated in the *O*-methylation of flavonoids or camptothecins in *C. acuminata* [13, 25]. However, the characterization of OMT genes responsible for flavonoid or camptothecin modification in *C. acuminata* remains limited.

In this study, we characterized the tissue-specific biosynthesis of these compounds through multi-omics analysis and functionally investigated a cluster of four *OMT* genes. Among them, CaOMT1, CaOMT2, and CaOMT3 were found to methylate both 9- and 10-hydroxycamptothecin. Furthermore, CaOMT1 and CaOMT3 are bifunctional, catalyzing the methylation of the 4′- and 7-hydroxyl groups of quercetin, respectively, while CaOMT2 is specific for the 4′-hydroxyl group. In contrast, CaOMT4 showed no detectable activity toward any of the tested substrates. Protein structure modeling and molecular docking studies revealed that residues F166, M313, and L316 were essential for the dual functionality of CaOMT3. Additionally, N261, S309, and T312 were identified as key amino acid residues that likely explained the absence of 7-*O*-methylation activity in CaOMT2. Furthermore, CaOMT4 showed no activity toward the tested substrates due to a 25-amino-acid deletion that impairs substrate binding. Collectively, our work refines the functional characterization of this *OMT* gene cluster and elucidates the molecular mechanism underlying the *O*-methylation of both hydroxycamptothecins and quercetin by these enzymes.

## Results

### Determination of metabolites and generation of transcriptomes in *C. acuminata*

*Camptotheca acuminata* produces abundant specialized metabolites, including flavonoids and alkaloids, which possess diverse pharmacological activities [31, 32]. We performed metabolomic profiling across seven tissues of *C. acuminata* and characterized the tissue-specific distribution of metabolites involved in representative pathways associated with quercetin and camptothecin (Figs S1–S5). Notably, tamarixetin and rhamnetin exhibited tissue-specific accumulation patterns enriched in stem phloem (Fig. S3). In contrast, glycosylated derivatives of quercetin, including quercetin-3′-*O*-glucuronide, accumulated in both leaves and stem phloem. The bioactive camptothecins, including camptothecin and methoxycamptothecin, were also predominantly localized in stem phloem, with quantified levels of 2.19 mg/g and 2.20 mg/g, respectively (Figs S4–S5). The accumulation patterns of these metabolites indicate that the enzymes responsible for the *O*-methylation of quercetin and camptothecin may exhibit preferential expression in the stem phloem.

To further investigate the underlying gene expression, we performed transcriptome sequencing of the same seven tissues. This generated 142.46 Gb of high-quality data, with over 94% of reads uniquely mapped to the reference genome (Tables S1–S3). A total of 28 060 unigenes were expressed, of which 94.28% were functionally annotated (Figs S6–S10). Additionally, principal component analysis confirmed distinct tissue identities and high reproducibility among the three biological replicates (Fig. S11). These results confirmed the high quality of the transcriptomic data generated in this study. The transcriptomic dataset has been deposited in the NCBI Sequence Read Archive (SRA) under accession PRJNA1377334, providing a foundation for elucidating tissue-specific secondary metabolism in *C. acuminata*.

### Differential transcriptome analysis of the camptothecin and flavonoid biosynthetic pathways

To analyze gene expression patterns across *C. acuminata* tissues, we examined 30 061 unigenes, among which 24 750 were expressed (TPM ≥ 1) in at least one tissue and 17 373 were expressed across all seven tissues examined (Fig. S12). The distribution of highly expressed unigenes (TPM > 1000) in each tissue was detailed in Data S1–S7. Based on the tissue-specific accumulation patterns of *O*-methylated camptothecin and quercetin, we conducted differential expression analysis by comparing the stem phloem with each of the other tissues individually (Figs S13–S16). The most significant difference was observed between stem phloem and mature leaf, with 10 373 differentially expressed genes (DEGs) identified (Fig. S14). Functional enrichment analysis suggested that the high number of DEGs in this comparison was largely driven by an enrichment in mature leaf of genes associated with ‘cellular processes’ (Fig. S17). Collectively, differential expression analysis provides a theoretical basis for screening candidate genes involved in the camptothecin and flavonoid biosynthesis pathways.

**Figure 1:**
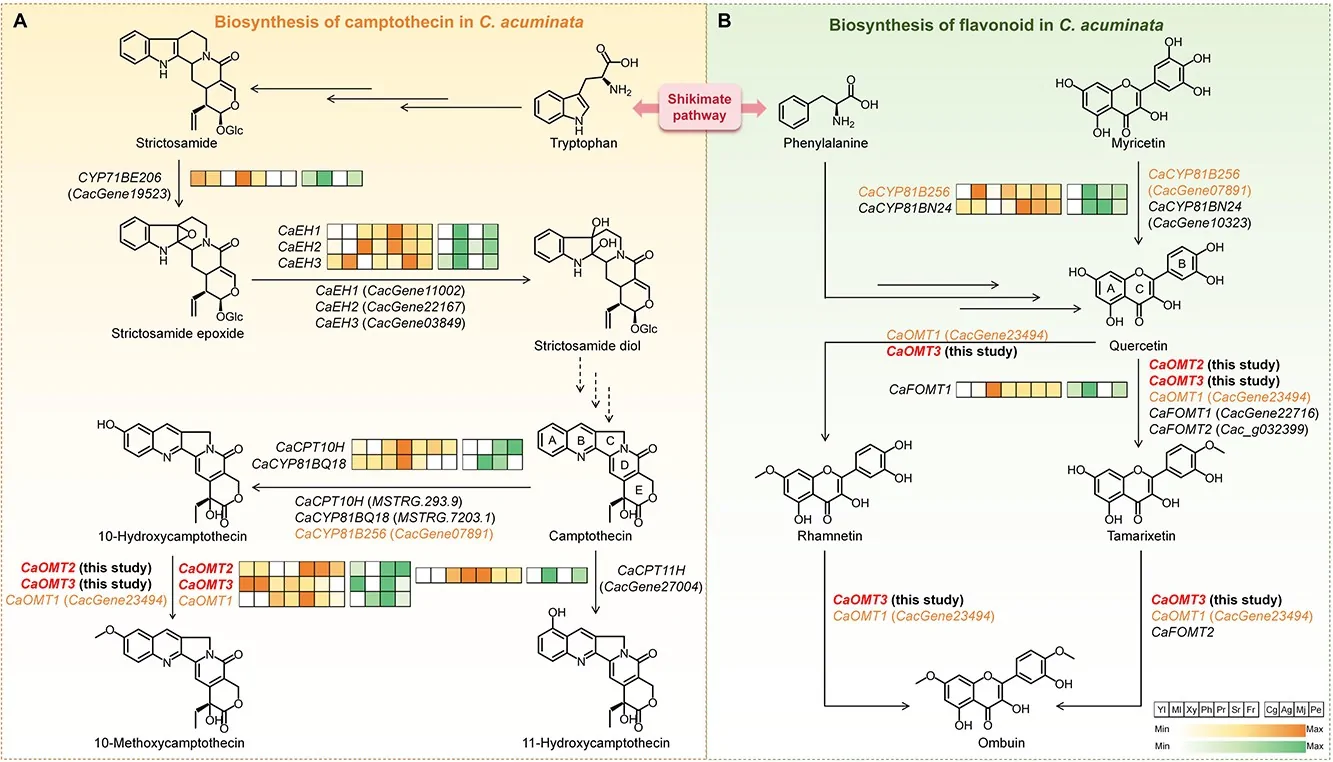
Advances in biosynthetic pathways of camptothecin and flavonoids in *C. acuminata*. (A) Biosynthesis of camptothecin in *C. acuminata*. *CYP*, *cytochrome P450*; *EH*, *epoxide hydrolase*; *CPT10H*, *camptothecin 10-hydroxylase*; *CPT11H*, *camptothecin 11-hydroxylase*; *OMT*, *O-methyltransferase*. (B) Biosynthesis of flavonoid in *C. acuminata*. *FOMT*, *flavonoid O-methyltransferase*. Green, validated enzymes in biosynthetic pathways of camptothecin or flavonoids in *C. acuminata*. Orange, validated enzymes in biosynthetic pathways of camptothecin and flavonoids in *C. acuminata*. Red, validated enzymes in the study. Solid arrow, characterized pathway steps; three arrows, multi-step reaction; dashed lines, unresolved pathway. An integrated heatmap showing the transcriptional levels of the genes was presented in the figure. Yl, young leaf; Ml, mature leaf; Xy, stem xylem; Ph, stem phloem; Pr, principal root; Sr, secondary lateral root; Fr, fibrous root; Cg, control group; Ag, AgNO_3_; Mj, MeJA; Pe, polyethylene glycol-20000.

To investigate the camptothecin biosynthetic pathway, we integrated transcriptome data from elicitor-treated and tissue-specific samples (Tables S4 and S5) [33]. We first investigated the expression patterns of genes involved in the biosynthetic pathway of camptothecin. Our analysis revealed distinct spatial expression patterns, with genes from the MVA and iridoid pathways being highly expressed in roots, whereas MEP pathway genes were predominantly expressed in leaves. Downstream biosynthetic genes were primarily localized in the primary root, stem phloem, or xylem and were found to be inducible under certain conditions (Fig. S18). In addition to transcriptomic insights, two high-quality *C. acuminata* genome assemblies are available to facilitate candidate gene screening. A previously identified camptothecin biosynthetic gene cluster includes a tandemly duplicated *10-hydroxygeraniol oxidoreductase*, a genomic feature that may contribute to metabolic diversification (Fig. S19A and Table S6) [34]. Furthermore, genomic analysis localized the *Ca10OMT* gene to a 106.3-kb scaffold harboring five genes linked to camptothecin biosynthesis (Fig. S19B and Table S7). This scaffold contains a *1-deoxy-D-xylulose 5-phosphate synthase* (involved in the MEP pathway) and four *O-methyltransferase* (*OMT*) genes. One of these OMTs has been functionally verified to catalyze the *O*-methylation of 10-hydroxycamptothecin, suggesting that the remaining three OMTs may perform analogous roles in the pathway (Fig. 1A). Integrated transcriptomic and genomic analyses revealed tissue-specific expression patterns and a clustered organization of camptothecin biosynthetic genes, providing a basis for candidate gene screening.

Flavonoids play essential roles in plant secondary metabolism [23, 24]. However, the biosynthesis of flavonoids in *C. acuminata* has remained unexplored for many years. Although a range of flavonoid compounds have recently been identified in this species, the expression patterns of flavonoid biosynthetic genes remain unclear [9]. Therefore, we analyzed genes implicated in the flavonoid biosynthesis pathway, using quercetin as a representative compound. In this study, a total of 38 unigenes involved in flavonoid biosynthesis were annotated (Fig. 1B and Fig. S20). Homologs of key backbone biosynthesis genes (*PAL*, *C4H*, and *4CL*) were highly expressed in all tissues (Fig. S20). In contrast, genes responsible for producing different flavonoid subtypes, such as *CHS*, *CHI*, *FNS*, *flavanone 3-hydroxylase* (*F3H*), and *flavanone 3′-hydroxylase* (*F3′H*), were predominantly highly expressed in aerial parts. Some genes involved in backbone modification, including hydroxylases and methyltransferases, were detected in stem xylem, phloem, and roots, and their expression could be induced by abiotic elicitors (Fig. 1B). The genes involved in backbone modification exhibit transcriptional expression patterns similar to those of genes involved in the camptothecin postmodification pathway. Collectively, our transcriptomic analysis provides a foundation for further exploration of the flavonoid biosynthesis pathway in *C. acuminata*.

### Functional characterization of the *CaOMT* gene cluster

**Figure 2:**
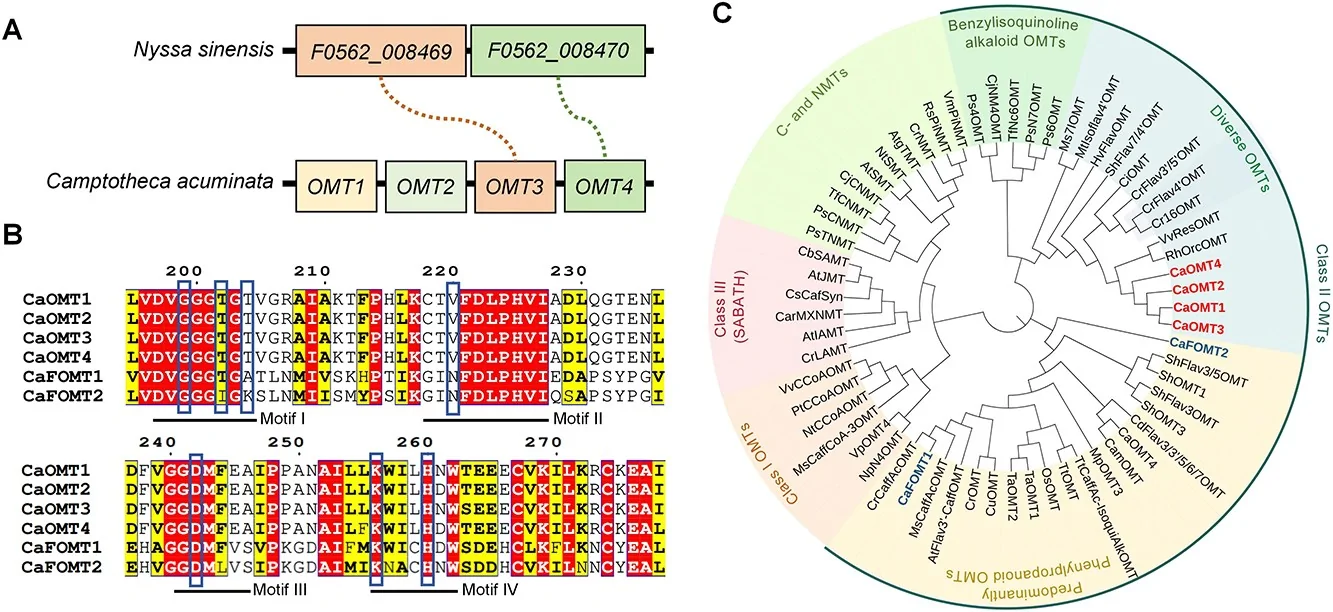
Synteny analysis, multiple sequence alignments, and phylogenetic tree of CaOMTs. (A) Cross-species synteny analysis of the *CaOMTs* gene cluster was performed between *C. acuminata* and its closely related species, *N. sinensis*. (B) Multiple sequence alignment of CaOMTs. Conserved motifs I–IV were underlined. The conserved residue was marked with blue boxes. (C) The phylogenetic tree of CaOMTs and other reported OMTs. The sequence data were provided in Table S10.

In *C. acuminata*, a wide range of oxygen-methylated derivatives have been identified, including 9- and 10-methoxycamptothecin from the camptothecin biosynthesis pathway, as well as ombuin and several others from the flavonoid biosynthesis pathway [9, 13, 35]. To date, only three OMT genes have been functionally identified in *C. acuminata*, with Ca10OMT exhibiting dual catalytic functions toward both alkaloid and flavonoid substrates [13]. However, the *OMT* gene family in *C. acuminata* remains largely unexplored. After integrating the published genome resources of *C. acuminata* and the transcriptome database constructed in this study and excluding redundant and incomplete sequences, a total of 40 *OMT* genes were identified (Table S8). Their physicochemical properties were listed in Table S8. At the chromosomal level, *Ca10OMT* (*CacGene23494*) was found to be located in a genomic locus adjacent to *CacGene23490*, *CacGene23491*, and *CacGene23492* (Fig. 2A and Table S7). Based on this genomic clustering, it was hypothesized that these three genes share similar OMT functions. Consequently, they were designated as *CaOMT2* (*CacGene23492*), *CaOMT3* (*CacGene23491*), and *CaOMT4* (*CacGene23490*), while *Ca10OMT* was assigned as *CaOMT1* (*CacGene23494*). To elucidate the evolutionary relationships within this *OMT* gene cluster, we conducted a comparative synteny analysis between species. This analysis revealed that only *CaOMT4* and *CaOMT3* possessed homologous genes in *Nyssaceae sinensis* (*F0562_008470* and *F0562_008469*, respectively) (Fig. S2A). We further performed phylogenetic reconstruction and Ka/Ks analysis (Fig. S21 and Table S9). The Ks values revealed a close relationship between CaOMT2 and CaOMT4, whereas phylogenetic analysis suggested a close relationship between CaOMT3 and CaOMT1. These results indicated that *CaOMT1* was derived from *CaOMT3*, and *CaOMT2* was derived from *CaOMT4*. Furthermore, all pairwise Ka/Ks ratios were <1, indicating that purifying selection has acted on these genes during evolution (Table S9).

Tissue-specific expression profiles showed that *CaOMT1*, *CaOMT2*, and *CaOMT4* were abundant in roots (primary and secondary lateral roots) (Fig. 1B and Fig. S22). In contrast, *CaOMT3* was specifically expressed in leaves. Notably, both *CaOMT1* and *CaOMT2* were induced by methyl jasmonate (MeJA). The correlation heatmap between methylated metabolites and the transcript levels of *CaOMTs* revealed that the expression level of *CaOMT3* was positively correlated with the accumulation of rhamnetin in tissues, suggesting its potential involvement in the *O*-methylation of quercetin (Fig. S23). Multiple sequence alignment revealed conserved features characteristic of the SAM-binding site in plant SAM-dependent methyltransferases, including the *S*-adenosylhomocysteine/*S*-adenosylmethionine (SAH/SAM) interaction region and four conserved motifs (I–IV) [28, 36] (Fig. 2B). Within these motifs, residues directly interacting with SAM, G199 in motif I, D222 in motif II, D242 in motif III, and K256 in motif IV, were conserved across the six CaOMTs examined. Moreover, H260 was present in all compared sequences. As a highly conserved residue essential for OMT catalysis, it typically forms a hydrogen bond with the hydroxyl group of the substrate [25, 28, 37]. Notably, specific amino acid substitutions were observed in key regions. For instance, the conserved GXGXGX sequence in motif I, which contains T204 in CaOMT1, CaOMT2, and CaOMT3, was replaced by A212 in CaFOMT1 and K218 in CaFOMT2. Similarly, T202 in CaOMT1, CaOMT2, and CaOMT3 was substituted by I216 in CaFOMT2. These variations reflect the functional diversity among CaOMTs, influencing their catalytic roles and substrate-binding conformations. In addition, a total of 56 OMT proteins derived from plants that are responsible for methylation were selected to conduct a phylogenetic analysis with the candidate OMTs (Fig. 2C and Table S10). Like CaOMT1, CaOMT2, CaOMT3, and CaOMT4 belong to the Class II subfamily of the diverse OMT clan, suggesting that these enzymes may be capable of catalyzing the *O*-methylation of both camptothecin derivatives and flavonoids.

### Expression and functional characterization of recombinant CaOMTs

**Figure 3:**
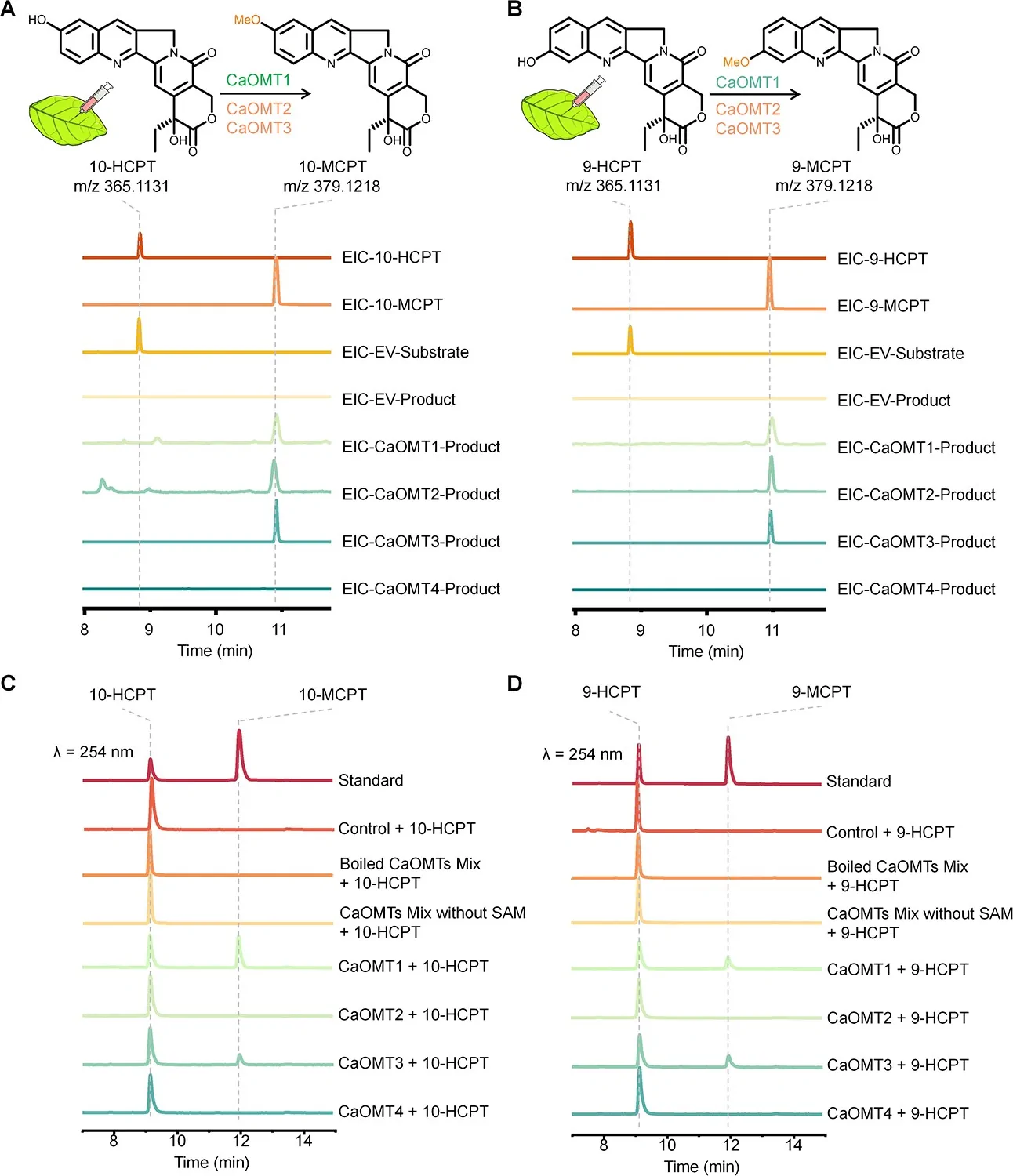
Catalytic analysis of CaOMTs with substrates 10-HCPT and 9-HCPT. (A) The extracted ion chromatograms (EICs) for the CaOMTs-harbored leaves co-infiltrated with 10-hydroxycamptothecin in *N. benthamiana*. (B) The EICs for the CaOMTs-harbored leaves co-infiltrated with 9-hydroxycamptothecin in *N. benthamiana*. (C) The results of HPLC for reactions of 10-hydroxycamptothecin with CaOMTs. Chromatograms were obtained at 254 nm wavelength. (D) The results of HPLC for reactions of 9-hydroxycamptothecin with CaOMTs. Chromatograms were obtained at 254 nm wavelength. 10-HCPT, 10-hydroxycamptothecin; 9-HCPT, 9-hydroxycamptothecin; 10-MCPT, 10-methoxycamptothecin; 9-MCPT, 9-methoxycamptothecin; CaOMTs Mix, mixed CaOMT proteins; SAM: S-adenosylmethionine.

*Camptotheca acuminata* contains not only 10-methoxycamptothecin but also 9-methoxycamptothecin [38, 39]. Previously, CaOMT1 was reported to exhibit bifunctional catalytic activity, mediating the *O*-methylation of 10-hydroxycamptothecin and quercetin. However, its ability to catalyze the *O*-methylation of the hydroxyl group at the C-9 position remains unknown. To investigate the functions of the *CaOMTs* gene cluster, we performed transient expression and catalytic assays in *Nicotiana benthamiana*. Agrobacterium strains harboring plant overexpression vectors *pHB-YFP* of the *CaOMT* genes were infiltrated into leaves. After two days, the substrates 9-hydroxycamptothecin and 10-hydroxycamptothecin were separately injected, and the samples were collected for analysis 3 days later. The methylase activity test indicated that the same mono-methylation product (m/z 379.1295) was detected in leaves expressing CaOMT1, CaOMT2, and CaOMT3, with a retention time matching that of the authentic standards for 9-methoxycamptothecin and 10-methoxycamptothecin (Fig. 3A and B). However, under physiological conditions in plant cells, no methylation activity was detectable for CaOMT4. Thus, we confirmed that CaOMT1, CaOMT2, and CaOMT3 exhibit methylase activity toward 10-hydroxycamptothecin and 9-hydroxycamptothecin *in planta*.

To further investigate their *O*-methylation activity *in vitro*, the *CaOMTs* genes were cloned into the *pCold-TF* vector and heterologously expressed in *Escherichia coli* BL21 (DE3). SDS-PAGE analysis showed that the recombinant CaOMTs were soluble in the intracellular supernatant (Fig. S24). The His-tagged CaOMT proteins were then purified to investigate their catalytic function. The assay used 9-hydroxycamptothecin and 10-hydroxycamptothecin as substrates. When 9-hydroxycamptothecin and 10-hydroxycamptothecin were used as substrates, new peaks corresponding to the *O*-methylated products were detected in the CaOMT1 and CaOMT3 protein reaction mixture by HPLC-DAD (Fig. 3C and D). UPLC-Q-TOF/MS analysis revealed that the molecular weight of the reaction products of CaOMT1 and CaOMT3 increased by 14.0143 Da (+ CH_2_), which was indicative of methylation. In addition, trace quantities of methoxycamptothecin were identified in the reaction products of CaOMT2 (Figs S25 and S26). Furthermore, both the retention time and mass spectrometry fragmentation patterns of the reaction product were identical to those of the methoxycamptothecin standard, with the fragment ions at m/z 335.1337 and 379.1295 showing perfect matches to the corresponding signals of the standard (Figs S27–S32) [2, 13]. In summary, CaOMT1, CaOMT2, and CaOMT3 are all capable of catalyzing the methylation of both 9- and 10-hydroxycamptothecin.

### Catalytic activity of CaOMTs on flavonoids

**Figure 4:**
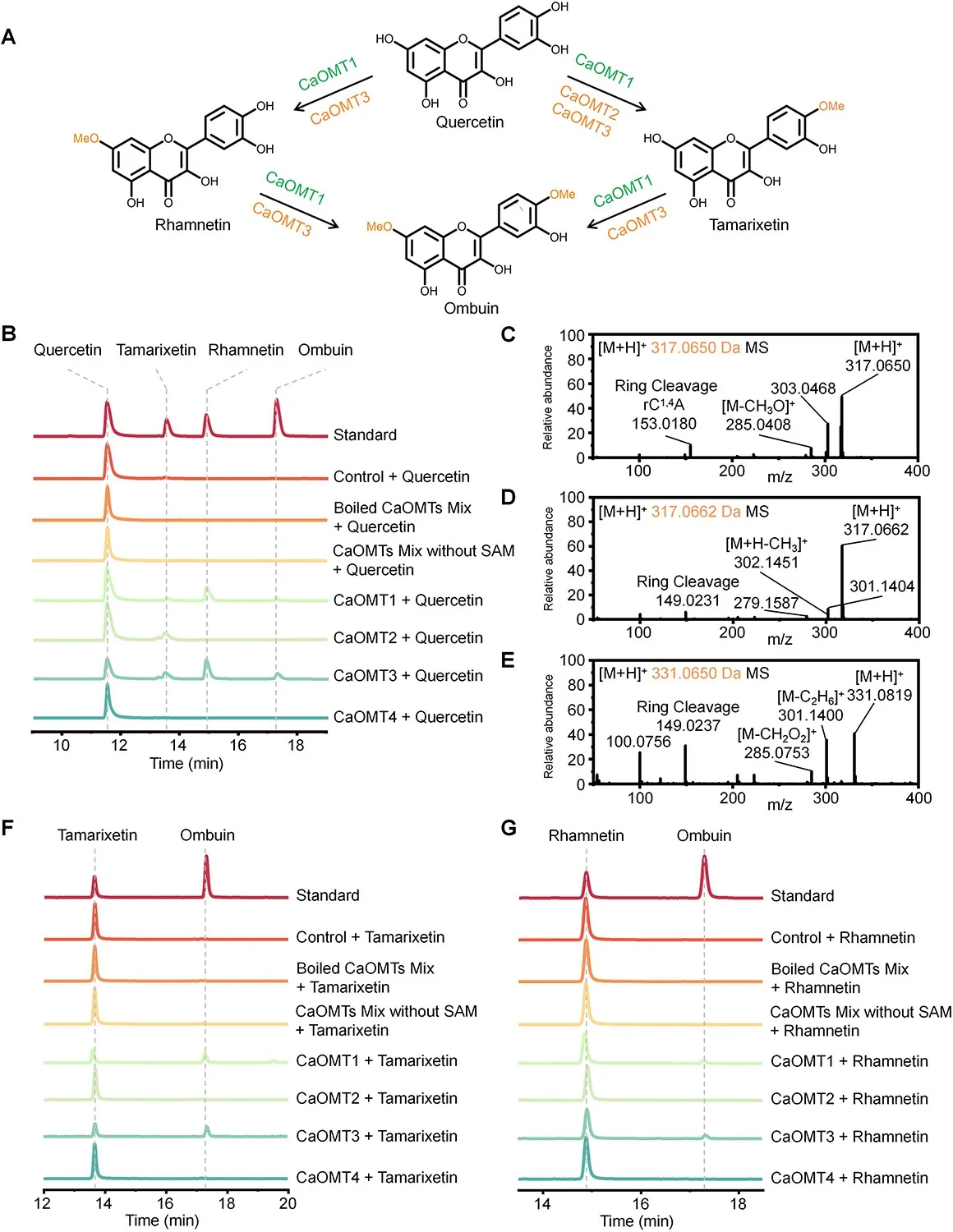
Catalytic activity of CaOMTs toward the substrates quercetin, tamarixetin, and rhamnetin. (A) Schematic summary of CaOMT catalysis using quercetin, tamarixetin, and rhamnetin as substrates. (B) HPLC analysis of quercetin reactions with CaOMTs. (C) Mass spectrum of the reaction product tamarixetin from the CaOMT1 reaction system. (D) Mass spectrum of the reaction product rhamnetin from the CaOMT1 reaction system. (E) Mass spectrum of the reaction product ombuin from the CaOMT1 reaction system. (F) HPLC analysis of tamarixetin reactions with CaOMTs. (G) HPLC analysis of rhamnetin reactions with CaOMTs. Chromatograms were monitored at 254 nm. CaOMTs Mix, mixture of CaOMT proteins; SAM: S-adenosylmethionine.

To further investigate the catalytic activity of CaOMTs on flavonoids, functional characterization was performed using quercetin as the reaction substrate. HPLC-DAD analysis revealed three distinct product peaks at retention times of 13.5 min, 14.8 min, and 17.2 min for CaOMT1 and CaOMT3, whereas only the peak at 13.5 min was observed for CaOMT2 (Fig. 4A and B). By comparison with authentic standards, these products were identified as tamarixetin (4′-*O*-methyl quercetin), rhamnetin (7-*O*-methyl quercetin), and ombuin (4′,7-dimethylquercetin), respectively, in order of increasing retention time. Further confirmation by UPLC-Q-TOF/MS showed that CaOMT1 and CaOMT3 produced two monomethylated derivatives (m/z 317.0650) and one dimethylated product (m/z 331.0819) (Figs S33 and S34). The compound eluting at 13.7 min was identified as tamarixetin based on its retention time and characteristic fragment ions at m/z 285.0408 and 153.0180 (Fig. 4C and Figs S35, S36). Similarly, the peak at 14.6 min matched rhamnetin in both retention time and diagnostic ion m/z 302.1451 (Fig. 4D and Fig. S37). The dimethylated product was confirmed as ombuin by co-elution with the standard and key fragments at m/z 301.1400 and 285.0753 (Fig. 4E and Fig. S38). In contrast, only tamarixetin was detected in the CaOMT2 reaction system (Fig. 4B and Fig. S35). To further investigate whether CaOMTs can also use monomethylated quercetin as a substrate to produce ombuin, enzymatic assays were performed with rhamnetin and tamarixetin. The results demonstrated that both CaOMT1 and CaOMT3 were able to catalyze the conversion of the monomethylated flavonoids, rhamnetin and tamarixetin, to ombuin (Fig. 4F and G). In contrast, CaOMT2 showed no activity toward rhamnetin and tamarixetin. Collectively, these results indicate that CaOMT1 and CaOMT3 catalyze the *O*-methylation of quercetin at both the 4′- and 7-hydroxyl groups, while CaOMT2 is specific to the 4′-position.

In addition, we tested other flavonoid substrates, including kaempferol. *In vitro* catalytic assays demonstrated that CaOMT1 and CaOMT3 could sequentially catalyze the *O*-methylation of kaempferol at the 7- and 4′-hydroxyl groups, yielding 3,5-dihydroxy-4′,7-dimethoxyflavone, whereas CaOMT2 exhibited no *O*-methylation activity toward kaempferol (Fig. S39). When kaempferide (kaempferol 4′-*O*-methyl ether) and rhamnocitrin (kaempferol 7-*O*-methyl ether) were used as substrates, CaOMT1 and CaOMT3 were also able to methylate them to generate 3,5-dihydroxy-4′,7-dimethoxyflavone (Figs S40 and S41). In contrast, CaOMT2 could not utilize kaempferide and rhamnocitrin as substrates. These results aligned with those obtained using quercetin as a substrate, indicating that CaOMT1 and CaOMT3 catalyze the *O*-methylation of kaempferol at both the 4′- and 7-hydroxyl groups.

### The optimum reaction conditions and kinetic parameters of CaOMTs

Using quercetin as a substrate, the optimal reaction temperature and pH for CaOMT1, CaOMT2, and CaOMT3 were determined. Both CaOMT1 and CaOMT3 exhibited an optimal pH of 8.0, while CaOMT2 showed optimal activity at pH 7.0 (Figs S42–S44). The optimal catalytic temperatures for CaOMT1, CaOMT2, and CaOMT3 were 45°C, 35°C, and 40°C, respectively (Figs S42–S44). The kinetic parameters were presented in Fig. S45. CaOMT1 (0.108 ± 0.001 mM) and CaOMT3 (0.120 ± 0.010 mM) exhibited comparable *K*m values, indicating their high affinity for quercetin. Meanwhile, CaOMT3 exhibited the highest *k*cat value, suggesting a faster turnover rate for quercetin. Furthermore, CaOMT3 exhibited the highest *k*cat/*K*m value of 7.54 ± 0.63 mM^−1^·s^−1^. These results indicated that CaOMT3 exhibited the highest catalytic efficiency toward quercetin among the three enzymes. Unlike CaOMT1 and CaOMT3, CaOMT2 displayed a significantly higher *K*m value (1.243 ± 0.197 mM), which was approximately ten times that of CaOMT3, indicative of its lower substrate affinity. Additionally, the *k*cat value of CaOMT2 was only (1.41 ± 0.14) × 10^−3^ s^−1^, suggesting a low turnover rate for quercetin. Furthermore, CaOMT2 exhibited a *k*cat/*K*m value of (4.75 ± 0.46) × 10^–5^ mM^−1^·s^−1^. Collectively, CaOMT1 and CaOMT3 showed higher catalytic activity than CaOMT2.

### Molecular insights into the substrate binding sites of CaOMTs

**Figure 5:**
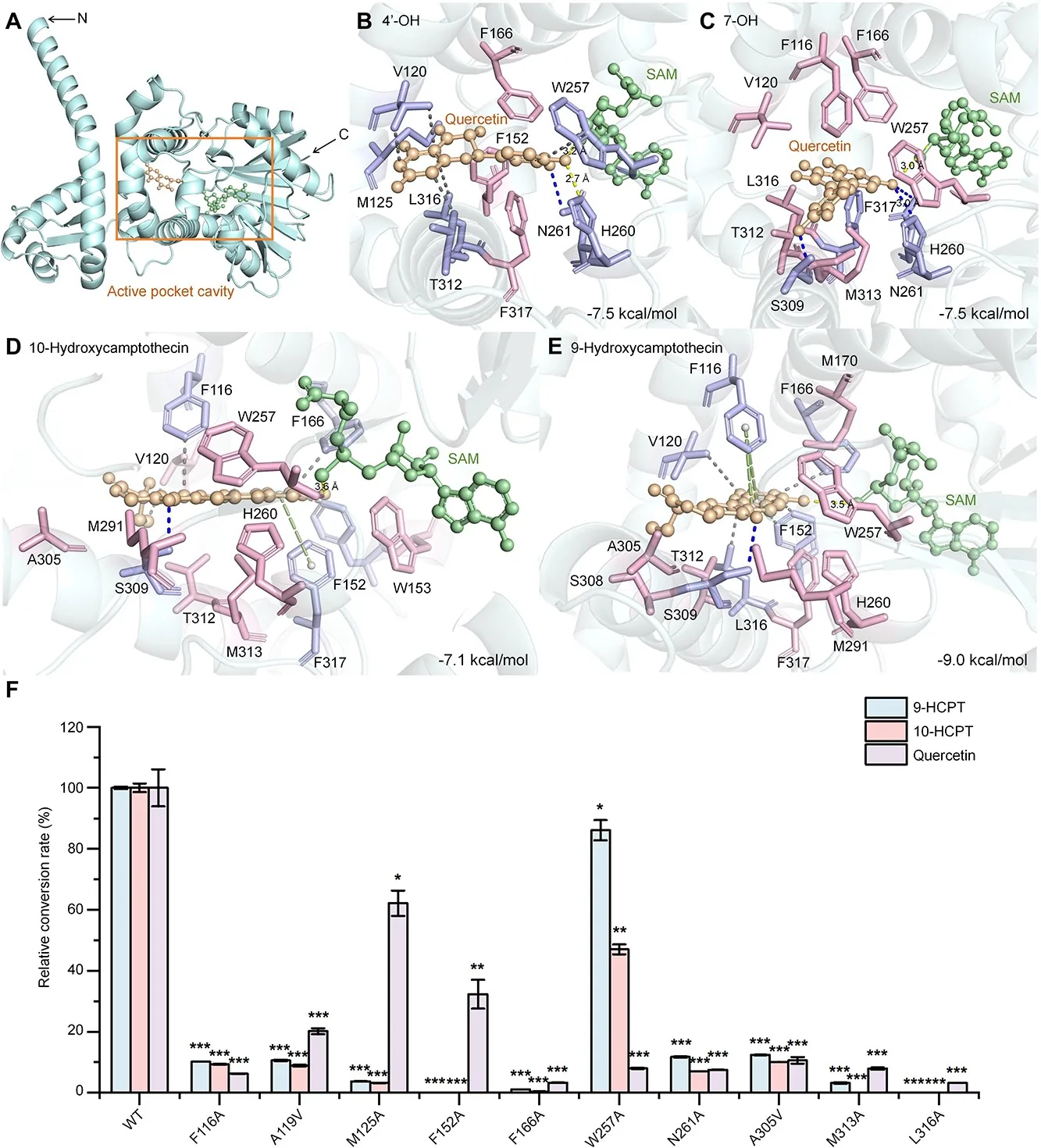
Three-dimensional structure analysis of CaOMT3 protein. (A) Overall architecture of CaOMT3 in complex with quercetin and SAM. (B) Amino acid residues surrounding the 4**′**-hydroxyl of quercetin in the binding pocket of CaOMT3. (C) Amino acid residues surrounding the 7-hydroxyl of quercetin in the binding pocket of CaOMT3. (D) Amino acid residues surrounding 10-hydroxycamptothecin bound in the binding pocket of CaOMT3. (E) Amino acid residues surrounding 9-hydroxycamptothecin bound in the binding pocket of CaOMT3. The binding free energy is shown in the lower right corner. (F) Catalytic activity of CaOMT3 mutants with substrates quercetin, 10-hydroxycamptothecin and 9-hydroxycamptothecin. Three biologically independent samples were tested (*n* = 3). All data represent the mean ± SD of three replicates. Student’s *t*-test: ^*^P <0.05; ^**^P <0.01; ^***^P <0.001.

To unveil the structural basis of the characterized CaOMTs, protein models were constructed using AlphaFold3, and quercetin substrate was docked into the binding pockets of CaOMT1, CaOMT2, and CaOMT3. The resulting structures with quercetin bound are shown in Fig. 5A–C and Figs S46, S47, revealing a similar spatial conformation upon binding the 4′-hydroxyl group of quercetin. Taking CaOMT3 as an example, multiple hydrophobic amino acids, including V120, M125, F152, F166, W257, M313, L316, and F317, surrounded the binding pocket cavity. When the 4′-hydroxyl group entered the cavity, V120, M125, W257, M313, and L316 engaged in hydrophobic interactions with the quercetin molecule (Fig. 5B). Simultaneously, N261 formed a hydrogen bond with the oxygen atom with the 3′-hydroxyl group of quercetin. This network of interactions positioned the SAM donor methyl group and the conserved catalytic residue H260 at distances of 3.2 Å and 2.7 Å, respectively, from the 4′-hydroxyl of quercetin, facilitating methylation. In contrast, when the 7-hydroxyl of quercetin entered the binding cavity, hydrogen bonds were formed between S309 and the 3′-hydroxyl and between N261 and H260 with the 7-hydroxyl group (Fig. 5C). This alternative binding mode maintained the SAM methyl group and H260 at distances of 3.6 Å and 3.0 Å, respectively, from the 7-hydroxyl of quercetin, enabling methylation at this position.

Furthermore, we docked 10-hydroxycamptothecin and 9-hydroxycamptothecin into the substrate-binding pockets of CaOMT1, CaOMT2, and CaOMT3, respectively (Fig. 5D, F, and Figs S48, S49). The docking revealed that, similar to the binding mode of quercetin, CaOMT3 formed hydrophobic interactions with both hydroxycamptothecins via surrounding hydrophobic residues, including F116, V120, F152, M291, and F317 (Fig. 5D and F). Additionally, in the complex structures of CaOMT3 with 9-hydroxycamptothecin and CaOMT1 with either 10- or 9-hydroxycamptothecin, F116 was observed to form π–π stacking interactions with the B-ring of hydroxycamptothecin, thereby stabilizing the ligand conformation (Fig. 5E and Fig. S48). In the binding conformations of CaOMT1 and CaOMT3 with hydroxycamptothecins, the distances between the oxygen atom of the 9- or 10-hydroxyl group and the methyl group of SAM were maintained at approximately 3.5 Å (Fig. 5D, E and Fig. S48). In contrast, for CaOMT2, these distances were 6.0 Å and 3.6 Å in complexes with 10-hydroxycamptothecin and 9-hydroxycamptothecin, respectively (Fig. S49). Meanwhile, the binding free energies of CaOMT2 with 10-hydroxycamptothecin and 9-hydroxycamptothecin were −5.9 kcal/mol and −2.6 kcal/mol, respectively. The relatively large distance between the substrate and SAM, together with the less favorable binding free energy, provides a plausible explanation for the low conversion rate of CaOMT2.

To confirm the functional significance of the predicted amino acid residues in CaOMT3, site-directed mutagenesis was performed (Fig. 5F and Fig. S50). Alanine substitution of hydrophobic residues, including F166, M313, and L316, reduced the relative conversion rates toward quercetin, 9-hydroxycamptothecin, and 10-hydroxycamptothecin by more than 90%, demonstrating that these residues are indispensable for catalysis (Fig. 5F). Furthermore, the activities of the F116A, A119V, N261A, and A305V mutants toward all three substrates decreased to less than 20% of the wild-type relative conversion rates. In addition, the M125A mutant exhibited a dramatic reduction in relative conversion rates for 9-hydroxycamptothecin and 10-hydroxycamptothecin to below 5%, while retaining 65.08% of its activity toward quercetin. This suggests that certain residues contribute more significantly to the catalysis of hydroxycamptothecins. Similarly, the W257A mutant retained 88.43% of its relative conversion rate for 9-hydroxycamptothecin, despite showing only 8.10% relative conversion rate toward quercetin. Collectively, these mutagenesis results indicate that a core set of hydrophobic residues, particularly F166, M313, and L316, is essential for the overall catalytic function of CaOMT3.

### Mechanism underlying the selectivity difference in quercetin *O*-methylation

**Figure 6:**
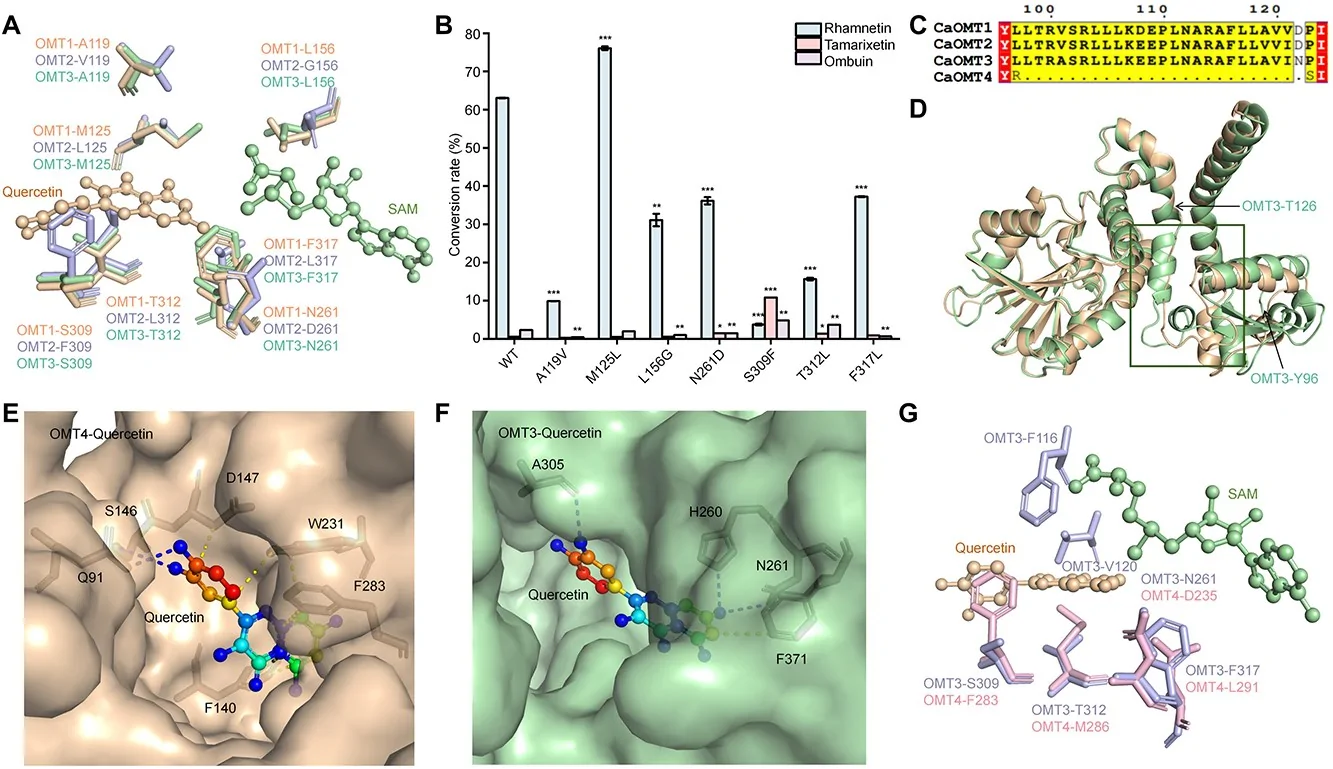
Comparison of the substrate-binding pockets of CaOMTs. (A) Comparison of differential sites among CaOMT1, CaOMT2, and CaOMT3. (B) Conversion rate of CaOMT3 mutants with substrate quercetin. Three biologically independent samples were tested (*n* = 3). Student’s *t*-test: ^*^P < 0.05; ^**^P < 0.01; ^***^P < 0.001. (C) Sequence alignment of CaOMTs showing a 25-amino-acid deletion and key residues in CaOMT4. (D) Overall structural comparison of the 3D protein structures of CaOMT3 and CaOMT4. (E) Surface representation of the substrate-binding pocket of CaOMT4 in complex with quercetin. (F) Surface representation of the substrate-binding pocket of CaOMT3 in complex with quercetin. (G) Comparison of amino acid residues surrounding quercetin within the binding pockets of CaOMT3 and CaOMT4.

Despite high sequence similarity among CaOMT1, CaOMT2, and CaOMT3, these enzymes exhibit distinct substrate selectivity. Specifically, CaOMT1 and CaOMT3 catalyze *O*-methylation of quercetin at both the 4′- and 7-hydroxyl groups, whereas CaOMT2 is specific to the 4′-position only. Binding free energy calculations support this observation. The CaOMT2 complex with the 7-hydroxyl group of quercetin showed a relatively high binding free energy (−1.3 kcal/mol), compared to −8.0 kcal/mol for CaOMT1 and −7.5 kcal/mol for CaOMT3 (Fig. 4B and Fig. S47). To elucidate the structural basis for this difference, we compared amino acid residues lining the substrate-binding pocket across the three enzymes (Fig. 6A). Seven residues were found to differ among the enzymes in close proximity to the ligand: A119, M125, L156, N261, S309, T312, and F317 in CaOMT1 and CaOMT3, which correspond to V119, L125, G156, D261, F309, L312, and L317 in CaOMT2, respectively. To determine whether these seven divergent residues account for the loss of 7-*O*-methylation activity in CaOMT2, we introduced individual CaOMT2-type substitutions into CaOMT3 (A119V, M125L, L156G, N261D, S309F, T312L, and F317L) and assessed their methylation activities (Fig. 6B). Functional assays revealed that, with the exception of M125L, all six remaining mutants exhibited significantly reduced conversion rates for quercetin to rhamnetin, explaining the lack of 7-*O*-methylation activity in CaOMT2. Notably, the S309F mutant displayed 20.30-fold and 2.08-fold higher conversion rates for quercetin to tamarixetin and ombuin, respectively, compared to wild type. Furthermore, N261D and T312L mutants also enhanced conversion rates toward 4′-*O*-methylated products. Collectively, D261, F309, and L312 in CaOMT2 (corresponding to N261, S309, and T312 in CaOMT1 and CaOMT3) govern the distinct regioselectivity of CaOMT2 toward quercetin 4′-*O*-methylation over 7-*O*-methylation.

### Absence of methylation activity in CaOMT4

The lack of methyltransferase activity in CaOMT4 toward the tested substrates can be attributed to multiple factors. Although CaOMT4 was expressed in a soluble form and purified successfully, proper folding could not be assumed a priori (Fig. S24). To assess its structural integrity, we performed circular dichroism (CD) spectroscopy on purified CaOMT4 (Fig. S51). The CD spectrum of native CaOMT4 displayed a characteristic minimum near 200 nm and featured two negative peaks at 208 and 222 nm, indicative of typical α-helical secondary structure. In contrast, heat-denatured CaOMT4 completely lost these spectral signatures. Notably, the CD profile of properly folded CaOMT3 closely resembled that of CaOMT4, confirming that the recombinant CaOMT4 adopted a well-ordered, native-like conformation. Furthermore, the loss of activity observed in tobacco-expressed CaOMT4 also suggested that its catalytic deficiency was not caused by protein misfolding (Fig. 4A and B).

Despite maintaining a folded structure, CaOMT4 harbors a 25-amino-acid deletion relative to other OMT family members, including CaOMT3 (Fig. 6C). This deletion encompasses a catalytically critical residue, A119. Mutation of this residue in CaOMT3 resulted in markedly reduced activity toward quercetin and hydroxylated camptothecin derivatives, underscoring its functional importance (Fig. 5F). To gain structural insights, we generated a homology model of CaOMT4 using AlphaFold3 and performed molecular docking with quercetin, 9-hydroxycamptothecin, and 10-hydroxycamptothecin (Fig. 6D–G and Figs S52, S53). When CaOMT4 was docked with quercetin, the substrate was able to approach the methyl group of SAM within the active site. However, the deletion of several surrounding hydrophobic residues (including F116 and V120 in CaOMT3) compromised stable substrate positioning within the binding pocket. These missing hydrophobic contacts likely destabilized the catalytically competent orientation of the substrate, thereby abolishing methylation activity (Fig. 6D–G). Furthermore, structural alignment revealed four residues near the substrate-binding pocket that differed between CaOMT4 and CaOMT3. Notably, the corresponding CaOMT3 mutants of these four residues have been demonstrated to exhibit decreased activity on quercetin (Fig. 6A, B, and  G). When CaOMT4 was docked with 9-hydroxycamptothecin and 10-hydroxycamptothecin, neither substrate was able to access the catalytic active site. Specifically, the hydroxyl groups of 9-hydroxycamptothecin and 10-hydroxycamptothecin were located 7.6 Å and 5.9 Å away from the methyl group of SAM, respectively, rendering CaOMT4 unable to catalyze the methylation of these hydroxycamptothecin derivatives (Fig. S53). Overall, the deletion of 25 amino acids resulted in the loss of key residues near the substrate-binding pocket and altered the overall pocket architecture. This structural change prevented the substrates from tightly binding or entering the pocket, ultimately leading to the loss of methylation activity toward the tested substrates.

## Discussion

Camptothecin, a natural product harvested from the stem phloem and seeds of *C. acuminata* and *Nothapodytes foetida*, has faced surging demand due to its derivatives irinotecan and topotecan [6, 40–42]. Although *C. acuminata* is the primary model for studying camptothecin biosynthesis, key secondary metabolic pathways remain largely uncharacterized. To date, several high-quality genome assemblies, along with multiple transcriptomes and proteomes, have been reported in *C. acuminata*, greatly facilitating the identification of candidate genes [21, 34, 39, 43–45]. These transcriptomic datasets were primarily generated from tissues at various developmental stages, including flowers, fruits, and seeds, based on seedlings with short growth periods and limited root differentiation [34, 45]. As a result, no distinction was made between taproots, fibrous roots, and lateral roots, despite substantial differences in gene expression patterns among these root types. In this study, we used 1-year-old plants with well-differentiated underground organs and simultaneously profiled the flavonoid and camptothecin biosynthetic pathways at both the metabolic and tissue-specific expression levels. The correlation between metabolite accumulation and spatial gene expression provides a valuable framework for identifying novel genes involved in camptothecin biosynthesis.

*O*-methylation is a key modification in the biosynthesis of various specialized metabolites, including phenylpropanoids, flavonoids, and alkaloids, altering their chemical properties and biological activities [25, 27, 28]. In *C. acuminata*, structurally similar methylated derivatives of flavonoids and camptothecins coexist within the same spatiotemporal context, suggesting the potential involvement of shared enzymes [9, 25]. In this study, we identified clustered *OMT* genes with similar sequences that are capable of catalyzing both the quercetin and the camptothecin derivatives 9-hydroxycamptothecin and 10-hydroxycamptothecin. Furthermore, it has been reported that CYP81B256 could hydroxylate camptothecin at the 10-position and quercetin at the C-5′-position [12].

Functional gene duplication is prevalent in *C. acuminata*. Numerous reported gene families involved in camptothecin biosynthesis have been found to contain two genes with similar sequences and identical functions, including two *SLAS* genes, two *7-DLH-like* genes, and several *cytochrome P450* genes [12, 46]. This phenomenon is likely attributable to whole-genome duplication events in *C. acuminata*. A total of 3213 collinear gene blocks and 33 904 pairs of collinear genes have been identified, spanning a total length of 315.1 Mb, which accounts for 84.60% of the genome assembly [43]. This suggests that a substantial portion of the genome has undergone duplication during evolution. In contrast to the previously reported duplicated genes such as *SLAS* and *7-DLH-like*, which typically retain identical functions, we observed that CaOMT2 lacks quercetin 7-*O*-methyltransferase activity, whereas CaOMT1 and CaOMT3 are capable of sequentially methylating the 7-hydroxyl and 4′-hydroxyl groups of quercetin. Based on this functional divergence, we performed protein homology modeling and molecular docking and identified seven amino acid residue differences located near the substrate-binding pocket of CaOMT2. Site-directed mutagenesis experiments further demonstrated that residues N261, S309, and T312 are critical for this differential catalytic activity.

## Conclusion

This study characterized the distribution patterns of flavonoids, camptothecin, and its derivatives across seven tissues of *C. acuminata* and generated corresponding tissue-specific transcriptomes. The biosynthetic pathways of two secondary metabolites (quercetin and camptothecin) in *C. acuminata* have been delineated by utilizing transcriptomic and genomic data from induced and tissue-specific samples. Concurrently, we analyzed the OMT gene family and screened four candidate genes based on their clustering in the genome. Biochemical assays demonstrated that CaOMT1, CaOMT2, and CaOMT3 were all capable of catalyzing the *O*-methylation of 9-HCPT and 10-HCPT. In addition, when the flavonoid quercetin was used as a substrate, CaOMT1 and CaOMT3 catalyzed *O*-methylation at both the 4′- and 7-hydroxyl groups, whereas CaOMT2 displayed strict regioselectivity, methylating exclusively the 4′-position. These findings expand the substrate spectrum of CaOMT1 to include the *O*-methylation of 9-hydroxycamptothecin. Additionally, CaOMT2 and CaOMT3 were newly identified as enzymes with dual *O*-methylation activity toward both quercetin and camptothecin derivatives. Key amino acid residues surrounding the substrate-binding pocket were identified through homology modeling and molecular docking, and their functional roles were preliminarily validated via site-directed mutagenesis. Among these, residues N261, S309, and T312 were determined to be critical for the lack of 7-hydroxyl methylation activity in CaOMT2. The 25-amino acid deletion in CaOMT4 alters the substrate-binding pocket architecture and results in the loss of some essential residues, thereby abolishing substrate binding and methylation activity. This study provides new insights into the functional diversity of OMTs involved in the biosynthesis of key secondary metabolites in *C. acuminata*.

## Materials and methods

### Transcriptome sequencing, *de novo* assembly, and functional annotation

Illumina-based deep transcriptome sequencing of the 21 samples was performed on the Illumina HiSeq 6000 platform (2 × 151 bp read length) by Shanghai Majorbio Bio-pharm Biotechnology Co., Ltd. (Shanghai, China). Subsequent data analysis was conducted using the Majorbio Cloud Platform [47, 48].

Following transcriptome sequencing, raw data from the 21 samples underwent quality control to produce clean reads. Adapters and low-quality sequences were trimmed using fastp. The clean reads were then aligned to the *C. acuminata* reference genome with hisat2, and mapping quality was assessed [43, 49]. Transcripts were assembled using stringtie, enabling the identification of novel transcripts through comparison with existing annotations [49]. These novel transcripts were subsequently functionally annotated. Throughout this process, reads mapping to exons were counted using RSeQC-2.3.6.

For functional annotation, all the unigenes were searched against six functional databases, including the NCBI nonredundant protein database, the Clusters of Orthologous Groups (COG), the Pfam, the Kyoto Encyclopedia of Genes and Genomes (KEGG), the Swiss-Prot, and the Gene Ontology database (GO) using BLASTX alignment with an E-value cut-off of 10^−5^. Subsequently, based on these alignments, the unigenes were functionally categorized using the GO, COG, and KEGG classification systems.

### Mining and screening of CaOMTs genes

The OMT family was identified in the *C. acuminata* genome and transcriptome using the *O*-methyltransferase domain (Pfam: PF00891; PF08100; PF04072; PF01135; PF03492; PF01596) using an E-value cutoff of <1e^−10^. Putative OMTs were further validated by confirming the presence of conserved domains via the NCBI Conserved Domain Database and SMART. Their theoretical isoelectric point (pI) and molecular weight (Mw) were predicted using the ExPASy Compute pI/Mw tool. Genomic analysis of *C. acuminata* identified a potential camptothecin biosynthetic gene cluster harboring candidate *OMT* genes [43]. The sequence alignment of conserved protein domains was performed using MEGA and visualized with ESPript 3.0. Multiple sequence alignment of the amino acids was performed with ClustalW in MEGA software, and a Neighbor-Joining phylogenetic tree was constructed with 1000 bootstrap replicates. The relevant sequences for the phylogenetic tree are presented in Table S10. In addition, the tree was annotated and visualized with iTOL.

### Interspecies collinearity analysis and Ka/Ks calculation of OMTs

Genome data for *N. sinensis* (accession numbers: SRX6441716, SRX6441715, SRX6441717, SRX6405746) and *C. acuminata* (accession numbers: PRJNA639006, SRR12042293, SRR12042292, SRR12042291) were downloaded from the NCBI SRA database. MCScanX (with default parameters) was employed to identify collinear gene pairs of *OMTs* between *C. acuminata* and *N. sinensis*. The synonymous substitution rates (Ks) for each gene within collinear gene pairs were calculated using the MCScanX downstream script add_ka_and_ks_to_collinearity.pl.

### Expression and catalysis of CaOMTs in *N. benthamiana*

Each *CaOMT* gene was cloned into the *pHB-YFP* vector at *Bam*HI and *Spe*I sites. Both the empty plasmid and the recombinant *pHB-CaOMT-YFP* construct were separately transformed into *Agrobacterium tumefaciens* GV3101. Transformed cells were grown in YEB medium to OD_600_ = 0.6, pelleted by centrifugation (4000 × g, 4°C, 5 min), and resuspended in infiltration buffer to the same density. For transient expression, suspensions were infiltrated into leaves of 5-week-old *N. benthamiana*. Leaves infiltrated with the empty vector served as the negative control. Substrates (100 μM 10-hydroxycamptothecin and 9-hydroxycamptothecin) were also infiltrated. After treatment, leaves were harvested, ground, and extracted with 500 μl methanol for 30 min. Extracts were filtered and analyzed by UPLC-Q-TOF/MS.

### Gene cloning, expression, and enzyme purification

Full-length candidate genes were amplified from cDNA using KOD FX High-Fidelity DNA polymerase (TOYOBO, Japan). The sequences of *CaOMT1*, *CaOMT2*, and *CaOMT3* were amplified from cDNA. The coding sequence of *CaOMT4* was synthesized by Zhejiang Shangya Biotechnology. For *CaOMT1*, *CaOMT2*, *CaOMT3*, and *CaOMT4*, the candidate genes were cloned into the *pCold-TF* vector via *Bam*HI/*Eco*RI sites using the ClonExpress II kit (Vazyme). The primer sequences are listed in Table S11. *Escherichia coli* Top10 was employed for plasmid construction, and *E. coli* BL21 (DE3) was utilized for recombinant protein production. Single colonies were inoculated into 10 ml LB medium and grown at 37°C for 12 h. Then, they were transferred to 200 ml fresh LB with ampicillin until OD_600_ reached 0.4. The protein was expressed by incubating with IPTG at 16°C for 12 h. The cells were harvested by centrifugation at 6000 × g for 10 min. Then, the cells were resuspended in PBS (20 mM potassium phosphate; pH 8.0; 100 mM NaCl; 5% glycerol) and lysed by sonication on ice. Following centrifugation at 6000 × g for 60 min, the supernatant was then loaded onto a Ni^2+^-resin column (Ni-NTA SefinoseTM Kit, Sangon Biotech). All purified proteins underwent dialysis, SDS-PAGE analysis, and buffer exchange. CaOMT1, CaOMT3, and CaOMT4 were dialyzed against PBS (pH 8.0), while CaOMT2 was dialyzed against PBS (pH 7.0). CD spectroscopy of CaOMT4 was performed using a Chirascan V100 CD spectrometer, and the data were analyzed with pro-Data Viewer software.

### CaOMTs protein enzymatic assay and methylation product identification

The stock solutions of quercetin, tamarixetin, rhamnetin, kaempferol, kaempferide, rhamnocitrin, 10-hydroxycamptothecin and 9-hydroxycamptothecin standard (50 mM in DMSO), and SAM (50 mM in H_2_O) were prepared (Shanghai Yuanye Bio-Technology Co., Ltd, Shanghai, China) and used for the enzymatic assay. The final reaction mixture (100 μl) contained 1 μl of substrate (50 mM), 2 μl of SAM (50 mM), and 50 μl of CaOMT recombinant protein (300 μg/ml) in PBS buffer (20 mM PBS, pH 8.0). The assay was performed at 37°C for 7 h. The negative control solution contained 1 μl of substrate (50 mM), 2 μl of SAM (50 mM), and 50 μl of empty *pCold-TF* vector protein (300 μg/ml) in a PBS buffer solution (20 mM, pH 8.0). After centrifugal drying, the reaction mixture was redissolved in 100 μl of methanol and filtered (0.22 μm). Each assay was performed in triplicate. The detection conditions for reaction products on HPLC-DAD were delineated in the preceding section. The methylation products were monitored using UPLC-Q-TOF/MS (Waters SYNAPT G2-Si, 2 μl injection) on a C18 column (2.1 × 50 mm, 1.7 μm) at 35°C. The mobile phase was a binary mixture of 0.1% formic acid in water (A) and acetonitrile (B). The gradient program was as follows: 0–2 min (5% B), 2–30 min (5%–100% B), 30–31 min (100% B), 31–33.5 min (100%–5% B), and 33.5–35 min (5% B). The flow rate was 0.3 ml/min. The MS experiment was conducted in a positive ion mode, with the following parameters: a capillary voltage of 2.5–3.0 kV, a cone voltage of 40 V, nitrogen as the drying gas, a desolvation gas flow of 800 l/h at 400°C, a cone gas flow of 50 l/h, a source temperature of 100°C, and a mass range of 50–1000 Da.

### Reaction condition optimization and kinetic characterization

HPLC-DAD was employed to prepare and analyze standard solutions of quercetin, tamarixetin, and ombuin. Single-factor experiments were carried out to identify the optimal reaction temperature and pH for each CaOMT. For kinetic analysis using quercetin as substrate, reaction mixtures were assembled with substrate concentrations ranging from 200 to 2000 μM and a fixed SAM concentration of 200 μM. All enzymatic reactions were conducted for 30 min under the previously established optimal conditions. Product formation rates were used to determine reaction velocities (V). The obtained velocity-substrate datasets were imported into Origin software, where nonlinear regression fitting to the Michaelis–Menten model was performed. Kinetic parameters, including the *V*max and *K*m, were subsequently generated by the software. The *k*cat was then derived by dividing *V*max by the enzyme concentration.

### Molecular docking and site-specific mutation

Three-dimensional models of CaOMT1–CaOMT4 were constructed using AlphaFold3. Ligand structures, including quercetin, 9- and 10-hydroxycamptothecin, and SAM, were obtained from PubChem. Docking calculations were performed with AutoDock 4.2.6, and the resulting complexes were analyzed in PyMOL (v2.5.0). Key residues for mutagenesis were identified as those within 4 Å of the docked substrates or exhibiting sequence divergence across the four enzymes. Corresponding mutagenic primers were provided in Table S11. Mutant CaOMT3 proteins were generated, expressed, and purified following previously described methods. Their catalytic efficiencies were evaluated by measuring reaction velocities and calculating relative activities under optimized conditions.

### Statistical analysis

All experiments in this study were repeated with at least three biological replicates. All data are presented as the mean ± SD. To test the difference between the control and treatment samples/transgenic hairy roots, a paired two-tailed Student’s *t*-test was used, and the significance threshold was *P* < 0.05.

## Supplementary Material

Web_Material_uhag145

## Data Availability

All data needed to support the conclusions in this paper are present in the paper and/or the supplementary materials.
