# Supplementary material for: The O-methyltransferase gene cluster in Camptotheca acuminata is involved in camptothecins and flavonoids metabolism
Source: Hortic Res. 2026 Apr 21;13(9):uhag145. doi: 10.1093/hr/uhag145 (PMC13503969; doi:10.1093/hr/uhag145)
Supplement: Supplementary file 1 [file web_material_uhag145.zip › Supplementary Figure.docx]

Supplementary Materials for

**The *O*-methyltransferase gene cluster in *Camptotheca acuminata* is involved in camptothecins and flavonoids metabolism**

Qingyan Ruan^1, #^, Xiaoxuan Fan^1, #^, Hao Zhan^1, #^, Maohua Cai^1^, Zengyuan Wang^1^, Qin Zhou^1^, Yue Feng^1^, Yiqing Peng^1^, Xiaolong Hao^1, *^, Guoyin Kai^1, *^

^1^ Laboratory of Medicinal Plant Biotechnology, School of Pharmaceutical Sciences, Zhejiang Chinese Medical University, Hangzhou 310053, China

^#^ These authors contributed equally to this work

* Corresponding Author

Prof. Dr. Guoyin Kai − Hangzhou, China, 310053; Email: kaiguoyin@zcmu.edu.cn; Tel/Fax: 86-0571-86633171.

Dr. Xiaolong Hao − Hangzhou, China, 310053; Email: haoxiaolong@zcmu.edu.cn; Tel/Fax: 86-0571-86633171.

Authors

Qingyan Ruan − Hangzhou, China, 310053; Email: 202211014011058@zcmu.edu.cn.

Xiaoxuan Fan − Hangzhou, China, 310053; Email: 202321114011066@zcmu.edu.cn.

Hao Zhan − Hangzhou, China, 310053; Email: 202321114011092@zcmu.edu.cn.

Maohua Cai − Hangzhou, China, 310053; Email: 202421014011122@zcmu.edu.cn.

Zengyuan Wang − Hangzhou, China, 310053; wangzengyuan@zcmu.edu.cn.

Qin Zhou − Hangzhou, China, 310053; 202521014011192@zcmu.edu.cn.

Yue Feng − Hangzhou, China, 310053; fengyue@zcmu.edu.cn.

Yiqing Peng − Hangzhou, China, 310053; 202321114011076@zcmu.edu.cn.

**Running title: *Camptotheca acuminata* OMT gene cluster**

**Figure S1 Principal component analysis (PCA) score plot derived from metabolic profiles.** PC1 and PC2 indicated the first and second principal components, with the percentages in parentheses representing the variance explained by each component.

**Figure S2 Heatmap visualization of the relative levels of flavonoids.** Yl, young leaf; Ml, mature leaf; Xy, stem xylem; Ph, stem phloem; Pr, principal root; Sr, secondary lateral root; Fr, fibrous root.

**Figure S3 Heatmap visualization of the relative levels of metabolites involved in the quercetin biosynthesis pathway.** The color scale represents normalized relative abundance (from low in green to high in orange). Yl, young leaf; Ml, mature leaf; Xy, stem xylem; Ph, stem phloem; Pr, principal root; Sr, secondary lateral root; Fr, fibrous root.

**Figure S4 Heatmap visualization of the relative levels of metabolites involved in the camptothecin biosynthesis pathway.** The color scale represents normalized relative abundance (from low in green to high in orange). Yl, young leaf; Ml, mature leaf; Xy, stem xylem; Ph, stem phloem; Pr, principal root; Sr, secondary lateral root; Fr, fibrous root.

**Figure S5 Schematic diagram of tissue sampling and quantitative analysis of camptothecins in *C. acuminata*.** (A) Seven tissues (young leaf, mature leaf, stem xylem, stem phloem, principal root, secondary lateral root and fibrous root) of one-year-old *C. acuminata* were used in this study for the metabolic analysis, as well as for transcriptome sequencing. (B) Contents of camptothecin, 10-hydroxycamptothecin, and 10-methoxycamptothecin across the seven tissues.

**Figure S6 The length distribution of the unigenes.** The x-axis represented the length of all assembled unigenes and the y-axis indicated the corresponding number of unigenes.

**Figure S7 Functional annotation of unigenes.** The left side of the figure displayed the number of unigenes annotated in each database. The upper part presented a bar chart illustrating the number of unique or shared unigenes among the six databases. The intersection among groups was displayed as highlighted points below the bar chart.

**Figure S8 COG classification chart.** The abscissa represented the functional classification of COG (expressed in Majuscule A ~ Z) and the y-axis represented the number of unigenes with such a function.

**Figure S9 Distributions of GO annotation of all unigenes.** The results were classified into three main categories: biological process, cellular component, and molecular function. The left y-axis indicated the percentage of a specific category of genes in that category. The right y-axis indicated the number of unigenes in a category.

**Figure S10 KEGG annotation and classification of unigenes.** The x-axis represented the number of unigenes and the y-axis represented the KEGG functional category.

**Figure S11 A principal component analysis (PCA) plot of transcriptomes from seven different tissues.** The x-axis represented principal component 1 (PC1), while the y-axis represented principal component 2 (PC2). Each dot in the plot denoted an individual sample, with different colors or shapes distinguishing sample groups. Samples within the same group were clustered closely together, indicating high reproducibility among biological replicates, whereas distinct separation was observed between different groups, reflecting significant differences in gene expression patterns induced by experimental treatments.

**Figure S12. Analysis of differentially expressed genes in seven tissues of *C. acuminata*.** (A) The Venn diagram revealed the number of expressed genes (TPM ≥ 1) in the samples. (B) The number of expressed genes (TPM ≥ 1) in each tissue was listed.

**Figure S13. Differentially expressed genes analysis of seven tissues in *C. acuminata*.** Differentially expressed unigenes profiling of six libraries of stem phloem versus fibrous root, stem phloem versus secondary lateral root, stem phloem versus principal root, stem phloem versus xylem, stem phloem versus mature leaf, stem phloem versus young leaf of *C. acuminata*. FDR < 0.05 and the absolute value of Log2FC ≥ 1 were used as the threshold to judge the significance of gene expression difference from transcriptome data.

**Figure S14. Volcano plot of expression differences.** (A) The volcano plot showed the differences in expression levels between stem phloem and young leaf. (B) The volcano plot showed the differences in expression levels between stem phloem and mature leaf.

**Figure S15. Volcano plot of expression differences.** (A) The volcano plot showed the differences in expression levels between stem phloem and xylem. (B) The volcano plot showed the differences in expression levels between stem phloem and principal root.

**Figure S16. Volcano plot of expression differences.** (A) The volcano plot showed the differences in expression levels between stem phloem and fibrous root. (B) The volcano plot showed the differences in expression levels between stem phloem and secondary lateral root.

**Figure S17. Gene Ontology (GO) enrichment analysis of differentially expressed genes between stem phloem and mature leaf.** The vertical axis showed GO terms, and the horizontal axis showed the Rich factor. Dot size indicated the number of unigenes in each GO term, and dot color represented the range of adjusted P-values (Padjust). A larger Rich factor signified a higher degree of enrichment.

**Figure 18 Transcriptional level annotation of the camptothecin biosynthesis upstream pathway.** MVA pathway, mevalonate pathway: *AACT*, *acetylCoA C-acetyltransferase*; *HMGS*, *3-hydroxy-3-methylglutaryl-CoA synthase*; *HMGR*, *3-hydroxy-3-methylglutaryl-CoA reductase*; *MK*, *mevalonate kinase*; *PMK*, *phosphomevalonate kinase*; *MDC*, *mevalonate 5-diphosphate decarboxylase*; *IDI*, *isopentenyl diphosphate isomerase*. MEP pathway, 2-C-methyl-D-erythritol 4-phosphate pathway: *DXS*, *1-deoxy-D-xylulose 5-phosphate synthase*; *DXR*, *1-deoxy-D-xylulose 5-phosphate reductoisomerase*; *CMS*, *4-(cytidine 5-diphospho)-2-C-methylerythritol synthase; CMK*, *4-(cytidine 5-diphospho)-2-C-methylerythritolkinase*; *MECS*, *2-C-methylerythritol-2,4-cyclodiphosphate synthase*; *HDS*, *hydroxymethylbutenyl 4-diphosphate synthase*; *HDR*, *1-hydroxy-2-methyl-butenyl 4-diphosphate reductase/hydroxymethylbutenyl diphosphate reductase*. Iridoid pathway: *GPPS*, *geranyl diphosphate synthase*; *GES*, *geraniol synthase*; *G10H*, *geraniol 10-hydroxylase*; *10-HGO*, *10-hydroxy-geraniol oxidoreductase*; *IS*, *iridoid synthase*; *ICYC*, *iridoid cyclase*; *IO*, *iridoid oxidase*; *7-DLGT*, *7-deoxyloganetic acid glucosyltransferase*; *7-DLH*, *7-deoxyloganic acid hydroxylase*; *SLAS*, *secologanic acid synthetase. Shikimate pathway*: *ASA*, *anthranilate synthase alpha*; *APT*, *anthranilate phosphoribosyltransferase*; *PAI*, *Phosphoribosylanthranilate isomerase*; *IGPS*, *Indole-3-glycerol-phosphate synthase*; *TS*, *tryptophan synthase*; *TDC*, *tryptophan decarboxylase*; *STRAS*, *strictosidinic acid synthase*. Yl, young leaf; Ml, mature leaf; Xy, stem xylem; Ph, stem phloem; Pr, principal root; Sr, secondary lateral root; Fr, fibrous root. Cg, control group; Ag, AgNO_3_; Mj, MeJA; Pe, polyethylene glycol-20000. Genes with validated functions were highlighted in orange.

**Figure S19. Physical clustering of genes involved in camptothecin biosynthetic pathways.** (A) A gene cluster identified as being involved in the biosynthesis of camptothecin. *10-HGO*, *10-hydroxygeraniol oxidoreductase*; *OMT*, *O-methyltransferase*; *7-DLH*, *7-deoxyloganic acid hydroxylase*; *IDI*, *isopentenyl diphosphate isomerase*. (B) A *10OMT*-containing gene cluster involved in camptothecin biosynthesis. *10OMT*, *10-hydroxycamptothecin O-methyltransferase*; *DXS*, *1-deoxy-D-xylulose 5-phosphate synthase*.

**Figure S20 Transcriptional level annotation of the flavonoid biosynthesis pathway.** *PAL*, *phenylalanine ammonia lyase*; *C4H*, *cinnamic acid 4-hydroxylase*; *4CL*, *4-coumarate: CoA ligase*; *ACCase*, *acetyl-CoA carboxylase*; *CHS*, *chalcone synthase*; *CHI*, *chalcone isomerase*; *F3H,* *flavanone 3-hydroxylase*; *F3′H*, *flavanone 3′-hydroxylase*; *FLS*, *flavonol synthase*. Yl, young leaf; Ml, mature leaf; Xy, stem xylem; Ph, stem phloem; Pr, principal root; Sr, secondary lateral root; Fr, fibrous root. Cg, control group; Ag, AgNO_3_; Mj, MeJA; Pe, polyethylene glycol-20000. Genes with validated functions were highlighted in orange.

**Figure S21 Phylogenetic tree of OMTs.** *F0562_008470* and *F0562_008469* from *Nyssa sinensis.* The tree was constructed using the neighbor-joining method, with a scale bar of 0.1 (indicating 0.1 substitutions per 100 residues )*.*

**Figure S22 Heatmap of *CaOMT4* gene transcript levels.** YL, young leaf; ML, mature leaf; XY, stem xylem; PH, stem phloem; PR, principal root; SR, secondary lateral root; FR, fibrous root. CG, control group; PEG, polyethylene glycol-20000.

**Figure S23 Heatmap showing the expression correlation of *CaOMT* genes with secondary metabolites (methylated flavonoids, methoxycamptothecin).** The hierarchical clustering analysis was performed based on the Spearman correlation coefficient between the expression levels of four *CaOMT* genes (*CaOMT1*, *CaOMT2*, *CaOMT3*, *CaOMT4*) and the accumulation of methylated flavonoids and methoxycamptothecin. The color scale ranges from −1 (blue, strong negative correlation) to 1 (red, strong positive correlation), with 0 representing no correlation.

**Figure S24 SDS-PAGE analysis of recombinant CaOMTs purified by Ni affinity chromatography.** (A) M, Protein marker. CaOMT1, CaOMT1 fusion protein with trigger factor. (B) M, Protein marker. CaOMT2, CaOMT2 fusion protein with trigger factor. (C) M, Protein marker. CaOMT3, CaOMT3 fusion protein with trigger factor. (D) M, Protein marker. CaOMT4, CaOMT4 fusion protein with trigger factor.

**Figure S25 Extracted ion chromatogram (m/z = 379.1295) analysis of 10-hydroxycamptothecin methylation by CaOMTs *in vitro*.** CaOMTs Mix: mixed CaOMT proteins; SAM: S-adenosylmethionine.

**Figure S26 Extracted ion chromatogram (m/z = 379.1295) analysis of 9-hydroxycamptothecin methylation by CaOMTs *in vitro*.** CaOMTs Mix: mixed CaOMT proteins; SAM: S-adenosylmethionine.

**Figure S27 Mass spectrometric data of the reaction product (10-MCPT) from the CaOMT1 assay.**

**Figure S28** **Mass spectrometric data of the reaction product (10-MCPT) from the CaOMT2 assay.**

**Figure S29 Mass spectrometric data of the reaction product (10-MCPT) from the CaOMT3 assay.**

**Figure S30 Mass spectrometric data of the reaction product (9-MCPT) from the CaOMT1 assay.**

**Figure S31 Mass spectrometric data of the reaction product (9-MCPT) from the CaOMT2 assay.**

**Figure S32 Mass spectrometric data of the reaction product (9-MCPT) from the CaOMT3 assay.**

**Figure S33 EICs for tamarixetin and rhamnetin ([M+H]^+^ = m/z 317.0650) were generated by UPLC-Q-TOF/MS analysis.** CaOMTs Mix: mixed CaOMT proteins; SAM: S-adenosylmethionine.

**Figure S34 EICs for ombuin ([M+H]^+^ = m/z 331.0650) were generated by UPLC-Q-TOF/MS analysis.** CaOMTs Mix: mixed CaOMT proteins; SAM: S-adenosylmethionine.

**Figure S35 Mass spectrometric data of the reaction product (tamarixetin) from the CaOMT2 assay.**

**Figure S36 Mass spectrometric data of the reaction product (tamarixetin) from the CaOMT3 assay.**

**Figure S37 Mass spectrometric data of the reaction product (rhamnetin) from the CaOMT3 assay.**

**Figure S38 Mass spectrometric data of the reaction product (ombuin) from the CaOMT3 assay.**

**Figure S39 HPLC analysis of kaempferol reactions with CaOMTs.** Chromatograms were detected at 254 nm. CaOMTs Mix: mixed CaOMT proteins; SAM: S-adenosylmethionine.

**Figure S40 HPLC analysis of kaempferide reactions with CaOMTs.** Chromatograms were detected at 254 nm. CaOMTs Mix: mixed CaOMT proteins; SAM: S-adenosylmethionine.

**Figure S41 HPLC analysis of rhamnocitrin reactions with CaOMTs.** Chromatograms were detected at 254 nm. CaOMTs Mix: mixed CaOMT proteins; SAM: S-adenosylmethionine.

**Figure S42 Enzymatic characterization of CaOMT1.** (A) Effects of reaction pH on enzyme activity of CaOMT1 with quercetin. pH 3-5: 20 mM HAc-NaAc, pH 6-8: 20 mM Na_2_HPO_4_-NaH_2_PO_4_, pH 9-10: 20 mM Tris-HCl. (B) Effects of reaction temperature on enzyme activity of CaOMT1 with quercetin.

**Figure S43 Enzymatic characterization of CaOMT2.** (A) Effects of reaction pH on enzyme activity of CaOMT2 with quercetin. pH 3-5: 20 mM HAc-NaAc, pH 6-8: 20 mM Na_2_HPO_4_-NaH_2_PO_4_, pH 9-10: 20 mM Tris-HCl. (B) Effects of reaction temperature on enzyme activity of CaOMT2 with quercetin.

**Figure S44 Enzymatic characterization of CaOMT3.** (A) Effects of reaction pH on enzyme activity of CaOMT3 with quercetin. pH 3-5: 20 mM HAc-NaAc, pH 6-8: 20 mM Na_2_HPO_4_-NaH_2_PO_4_, pH 9-10: 20 mM Tris-HCl. (B) Effects of reaction temperature on enzyme activity of CaOMT3 with quercetin.

**Figure S45** **The catalytic kinetic parameters of CaOMTs with quercetin as substrate.** (A) The catalytic kinetic parameters of CaOMT1 with quercetin as substrate. (B) The catalytic kinetic parameters of CaOMT2 with quercetin as substrate. (C) The catalytic kinetic parameters of CaOMT3 with quercetin as substrate.

**Figure S46. Three-dimensional structure analysis of CaOMT1 protein with quercetin.** (A) Amino acid residues surrounding the 4'-OH group of quercetin within the binding pocket of CaOMT1. (B) Amino acid residues surrounding the 7-OH group of quercetin within the binding pocket of CaOMT1. Blue dashed lines represent hydrogen bonding interactions, while grey dashed lines denote hydrophobic interactions.

**Figure S47. Three-dimensional structure analysis of CaOMT2 protein with quercetin.** (A) Amino acid residues surrounding the 4'-OH group of quercetin within the binding pocket of CaOMT2. (B) Amino acid residues surrounding the 7-OH group of quercetin within the binding pocket of CaOMT2. Blue dashed lines represent hydrogen bonding interactions, while grey dashed lines denote hydrophobic interactions.

**Figure S48. Three-dimensional structure analysis of CaOMT1 protein with hydroxycamptothecin.** (A) Amino acid residues surrounding the 10-hydroxycamptothecin within the binding pocket of CaOMT1. (B) Amino acid residues surrounding the 9-hydroxycamptothecin within the binding pocket of CaOMT1. Green dashed lines represent π-π stacking interactions, while grey dashed lines denote hydrophobic interactions.

**Figure S49. Three-dimensional structure analysis of CaOMT2 protein with hydroxycamptothecin.** (A) Amino acid residues surrounding the 10-hydroxycamptothecin within the binding pocket of CaOMT2. (B) Amino acid residues surrounding the 9-hydroxycamptothecin within the binding pocket of CaOMT2. Blue dashed lines represent hydrogen bonding interactions, while grey dashed lines denote hydrophobic interactions.

**Figure S50. Catalytic activity of CaOMT3 mutants with substrates quercetin, 10-hydroxycamptothecin and 9-hydroxycamptothecin.** Three biologically independent samples were tested (n=3). All data represent the mean ± SD of three replicates. Student’s t-test: **p* <0.05; ***p* < 0.01; ****p* < 0.001.

**Figure S51 Circular dichroism spectrum of CaOMT4.** The spectrum was recorded at 25^o^C in 20 mM phosphate buffer (pH 8.0). Boiled CaOMT4 served as the negative control, while CaOMT3 served as the positive control.

**Figure S52. Three-dimensional structure analysis of CaOMT4 protein with quercetin.** (A) Amino acid residues surrounding the 4'-OH group of quercetin within the binding pocket of CaOMT4. (B) Amino acid residues surrounding the 7-OH group of quercetin within the binding pocket of CaOMT4.

**Figure S53 Three-dimensional structure analysis of CaOMT4 protein with hydroxycamptothecin.** (A) Amino acid residues surrounding the 10-hydroxycamptothecin within the binding pocket of CaOMT4. (B) Amino acid residues surrounding the 9-hydroxycamptothecin within the binding pocket of CaOMT4.
