# Supplementary material for: The O-methyltransferase gene cluster in Camptotheca acuminata is involved in camptothecins and flavonoids metabolism
Source: Hortic Res. 2026 Apr 21;13(9):uhag145. doi: 10.1093/hr/uhag145 (PMC13503969; doi:10.1093/hr/uhag145)
Supplement: Supplementary file 1 [file web_material_uhag145.zip › Supplementary Methods.docx]

Supplementary Materials for

**The *O*-methyltransferase gene cluster in *Camptotheca acuminata* is involved in camptothecins and flavonoids metabolism**

Qingyan Ruan^1, #^, Xiaoxuan Fan^1, #^, Hao Zhan^1, #^, Maohua Cai^1^, Zengyuan Wang^1^, Qin Zhou^1^, Yue Feng^1^, Yiqing Peng^1^, Xiaolong Hao^1, *^, Guoyin Kai^1, *^

^1^ Laboratory of Medicinal Plant Biotechnology, School of Pharmaceutical Sciences, Zhejiang Chinese Medical University, Hangzhou 310053, China

^#^ These authors contributed equally to this work

* Corresponding Author

Prof. Dr. Guoyin Kai − Hangzhou, China, 310053; Email: kaiguoyin@zcmu.edu.cn; Tel/Fax: 86-0571-86633171.

Dr. Xiaolong Hao − Hangzhou, China, 310053; Email: haoxiaolong@zcmu.edu.cn; Tel/Fax: 86-0571-86633171.

Authors

Qingyan Ruan − Hangzhou, China, 310053; Email: 202211014011058@zcmu.edu.cn.

Xiaoxuan Fan − Hangzhou, China, 310053; Email: 202321114011066@zcmu.edu.cn.

Hao Zhan − Hangzhou, China, 310053; Email: 202321114011092@zcmu.edu.cn.

Maohua Cai − Hangzhou, China, 310053; Email: 202421014011122@zcmu.edu.cn.

Zengyuan Wang − Hangzhou, China, 310053; wangzengyuan@zcmu.edu.cn.

Qin Zhou − Hangzhou, China, 310053; 202521014011192@zcmu.edu.cn.

Yue Feng − Hangzhou, China, 310053; fengyue@zcmu.edu.cn.

Yiqing Peng − Hangzhou, China, 310053; 202321114011076@zcmu.edu.cn.

**Running title: *Camptotheca acuminata* OMT gene cluster**

**Supplementary Methods**

**Plant materials**

Seven different tissues (young leaf, mature leaf, stem xylem, stem phloem, principal root, secondary lateral root and fibrous root) were collected from one-year-old *C. acuminata* plants grown in a greenhouse at Zhejiang Chinese Medical University (Fig. S5A). Each tissue included three biological replicates. Plants were cultivated under controlled conditions: 25 ± 2^o^C, with a 16-hour light/8-hour dark cycle. Twenty-one samples intended for metabolite detection and RNA extraction were collected, and then immediately frozen in liquid nitrogen and stored in a -80^o^C freezer.

**Determination of compounds in *C. acuminata***

In the present study, untargeted profiling of various metabolite classes, including flavonoids, was conducted. Twenty-one samples were extracted from 50 mg sample powder with 400 µL methanol: water (4:1, v/v) containing internal standards, ultrasonicated at 5^o^C for 30 min, incubated at -20^o^C for 30 min, and centrifuged at 13,000 g for 15 min at 4^o^C. The supernatant was analyzed via UHPLC-Triple TOF (AB SCIEX) using an ACQUITY UPLC HSS T3 column (100 mm × 2.1 mm, 1.8 µm) at 45^o^C. Mobile phases A (95% water/5% acetonitrile + 0.1% formic acid) and B (47.5% acetonitrile/47.5% isopropanol/5% water + 0.1% formic acid) were delivered at 0.40 mL/min; injection volume 10 µL. MS data were acquired in positive/negative ion modes over m/z 50-1,000. Source conditions: ion spray voltage + 5500/-4500 V, source temperature 500^o^C, gas1/gas2 50 psi, curtain gas 35 psi; DP 80 V, CE 40 ± 20 eV.

Raw data were processed using Progenesis QI (Waters Corporation, Milford, USA) for peak detection, extraction, alignment, and integration. Compound annotation was performed using the HMDB and Metlin databases. To minimize or eliminate variations introduced during the experiment and data analysis, the identified features underwent preprocessing. Features with missing values exceeding 20% within any group were removed from the raw data. The remaining missing values were imputed using the minimum value detected across all samples. The response intensities of the mass spectral peaks were normalized using the sum intensity normalization method to obtain a normalized data matrix. Variables with a relative standard deviation (RSD) greater than 30% in quality control (QC) samples were removed. Finally, the data were log_10_-transformed, resulting in the final data matrix for subsequent analysis. Statistical analyses, including PCA, were performed using the ropls package (Version 1.6.2) in R. The integration of metabolomic and transcriptomic data was conducted using Spearman's rank correlation coefficient, and the results were visualized as a heatmap.

To quantify camptothecin, 10-hydroxycamptothecin, and 10-methoxycamptothecin in seven tissues of *C. acuminata* , samples were ground in liquid nitrogen and freeze-dried to constant weight (Labconco, USA). Standards were obtained from Shanghai Yuanye Bio-Technology Co., Ltd. Powdered samples (50 mg) were ultrasonically extracted with 1 mL methanol for 30 min, then centrifuged at 12,000 rpm for 10 min at room temperature. The supernatants were collected for HPLC analysis. Chromatographic separation was performed on an Agilent 1260 Infinity II system with a DAD detector using a COSMOSIL 5C18-AR-II column (3.5 μm, 4.6 × 150 mm). The mobile phase consisted of acetonitrile (A) and 0.1% formic acid in water (B) with a gradient: 0–25 min, 30% A; 25–30 min, 100% A; 30–35 min, 30% A. The flow rate was 0.8 mL/min, injection volume was 5 μL, and detection was at 254 nm.

**RNA extraction**

Total RNA was isolated from approximately 150 mg of *C. acuminata* tissues with the RNAprep Pure Plant Kit (Tiangen, China), incorporating an on-column DNase I digestion. The isolated RNA was quantified using NanoDrop One (Thermo Scientific™, USA), and its integrity was confirmed by gel electrophoresis. First-strand cDNA was synthesized from 1 µg RNA using the FastKing RT Kit (with gDNase, Tiangen, China).

**Differential expression analysis of unigenes**

Gene expression levels were quantified as TPM (Transcripts Per Million) values to enable cross-sample comparison. Briefly, clean reads from all 21 transcriptome libraries were mapped to the assembled unigenes using Bowtie, and TPM values for each unigene were calculated with RSEM. Differential expression analysis was then performed by comparing the stem phloem tissue against each of the other six tissues. Genes meeting the criteria of |Fold Change| ≥ 2 and a FDR (False Discovery Rate) < 0.05 were identified as differentially expressed genes (DEGs).

**De novo reassembly and characterization of the induced transcriptome from *C. acuminata***

The raw transcriptome data for *C. acuminata* were obtained from the NCBI BioProject database under accession number PRJNA704189. The clean reads were aligned to the reference genome, and the quality of this alignment was assessed. Subsequent analyses were performed as described in the previous sections.
