# Supplementary material for: The O-methyltransferase gene cluster in Camptotheca acuminata is involved in camptothecins and flavonoids metabolism
Source: Hortic Res. 2026 Apr 21;13(9):uhag145. doi: 10.1093/hr/uhag145 (PMC13503969; doi:10.1093/hr/uhag145)
Supplement: Supplementary file 1 [file web_material_uhag145.zip › Supplementary Table.docx]

Supplementary Materials for

**The *O*-methyltransferase gene cluster in *Camptotheca acuminata* is involved in camptothecins and flavonoids metabolism**

Qingyan Ruan^1, #^, Xiaoxuan Fan^1, #^, Hao Zhan^1, #^, Maohua Cai^1^, Zengyuan Wang^1^, Qin Zhou^1^, Yue Feng^1^, Yiqing Peng^1^, Xiaolong Hao^1, *^, Guoyin Kai^1, *^

^1^ Laboratory of Medicinal Plant Biotechnology, School of Pharmaceutical Sciences, Zhejiang Chinese Medical University, Hangzhou 310053, China

^#^ These authors contributed equally to this work

* Corresponding Author

Prof. Dr. Guoyin Kai − Hangzhou, China, 310053; Email: kaiguoyin@zcmu.edu.cn; Tel/Fax: 86-0571-86633171.

Dr. Xiaolong Hao − Hangzhou, China, 310053; Email: haoxiaolong@zcmu.edu.cn; Tel/Fax: 86-0571-86633171.

Authors

Qingyan Ruan − Hangzhou, China, 310053; Email: 202211014011058@zcmu.edu.cn.

Xiaoxuan Fan − Hangzhou, China, 310053; Email: 202321114011066@zcmu.edu.cn.

Hao Zhan − Hangzhou, China, 310053; Email: 202321114011092@zcmu.edu.cn.

Maohua Cai − Hangzhou, China, 310053; Email: 202421014011122@zcmu.edu.cn.

Zengyuan Wang − Hangzhou, China, 310053; wangzengyuan@zcmu.edu.cn.

Qin Zhou − Hangzhou, China, 310053; 202521014011192@zcmu.edu.cn.

Yue Feng − Hangzhou, China, 310053; fengyue@zcmu.edu.cn.

Yiqing Peng − Hangzhou, China, 310053; 202321114011076@zcmu.edu.cn.

**Running title: *Camptotheca acuminata* OMT gene cluster**

**Table S1 Summary of transcriptomes from seven different tissues in *C. acuminata*.**

| Sample | Raw reads | Raw bases | Clean reads | Clean bases | Error rate(%) | Q20(%) | Q30(%) | GC content(%) |
| --- | --- | --- | --- | --- | --- | --- | --- | --- |
| Young leaf1 | 46,399,816 | 7,006,372,216 | 46,078,916 | 6,766,991,217 | 0.0251 | 97.99 | 94.1 | 44.71 |
| Young leaf2 | 46,536,378 | 7,026,993,078 | 46,144,530 | 6,797,534,093 | 0.0254 | 97.87 | 93.77 | 44.78 |
| Young leaf3 | 55,065,932 | 8,314,955,732 | 54,696,468 | 8,055,780,609 | 0.0249 | 98.04 | 94.21 | 44.83 |
| Mature leaf1 | 43,925,540 | 6,632,756,540 | 43,638,108 | 6,456,023,915 | 0.025 | 98.02 | 94.14 | 44.48 |
| Mature leaf2 | 47,952,206 | 7,240,783,106 | 47,662,858 | 7,056,088,857 | 0.025 | 98.04 | 94.17 | 44.69 |
| Mature leaf3 | 45,485,138 | 6,868,255,838 | 45,185,150 | 6,663,366,833 | 0.025 | 98.01 | 94.14 | 44.78 |
| Stem xylem1 | 43,533,182 | 6,573,510,482 | 43,241,248 | 6,390,369,567 | 0.0252 | 97.95 | 93.98 | 44.61 |
| Stem xylem2 | 45,988,640 | 6,944,284,640 | 45,714,492 | 6,742,768,164 | 0.0248 | 98.1 | 94.3 | 44.59 |
| Stem xylem3 | 47,882,886 | 7,230,315,786 | 47,510,830 | 6,973,954,538 | 0.025 | 98.01 | 94.17 | 44.83 |
| Stem phloem1 | 45,636,748 | 6,891,148,948 | 45,306,012 | 6,661,534,133 | 0.0251 | 97.97 | 94.06 | 44.33 |
| Stem phloem2 | 45,768,228 | 6,911,002,428 | 45,437,264 | 6,668,048,282 | 0.0252 | 97.96 | 93.97 | 44.37 |
| Stem phloem3 | 48,301,250 | 7,293,488,750 | 47,900,044 | 6,993,008,981 | 0.0252 | 97.94 | 93.99 | 44.69 |
| Principal root1 | 45,006,512 | 6,795,983,312 | 44,695,002 | 6,538,322,046 | 0.0249 | 98.05 | 94.28 | 44.46 |
| Principal root2 | 46,566,800 | 7,031,586,800 | 46,191,980 | 6,781,894,306 | 0.0253 | 97.89 | 93.85 | 44.35 |
| Principal root3 | 45,402,294 | 6,855,746,394 | 44,956,244 | 6,607,629,278 | 0.0255 | 97.79 | 93.65 | 44.57 |
| Secondary lateral root1 | 48,203,168 | 7,278,678,368 | 47,828,434 | 7,052,454,578 | 0.0251 | 97.96 | 94.08 | 44.32 |
| Secondary lateral root2 | 43,177,594 | 6,519,816,694 | 42,857,354 | 6,286,401,057 | 0.025 | 97.99 | 94.18 | 44.24 |
| Secondary lateral root3 | 43,700,466 | 6,598,770,366 | 43,332,426 | 6,344,487,946 | 0.025 | 97.99 | 94.14 | 44.48 |
| Fibrous root1 | 46,944,868 | 7,088,675,068 | 46,574,578 | 6,843,454,464 | 0.0251 | 97.98 | 94.12 | 44.55 |
| Fibrous root2 | 44,899,058 | 6,779,757,758 | 44,387,462 | 6,523,000,587 | 0.0259 | 97.65 | 93.34 | 44.56 |
| Fibrous root3 | 49,550,116 | 7,482,067,516 | 49,119,890 | 7,252,675,037 | 0.0253 | 97.9 | 93.9 | 44.47 |

**Table S2 Summary of transcriptome and reference genome alignment results.**

| Sample | Total reads | Total mapped | Multiple mapped | Uniquely mapped |
| --- | --- | --- | --- | --- |
| Young leaf1 | 46,078,916 | 43,839,296 (95.14%) | 1,138,961 (2.47%) | 42,700,335 (92.67%) |
| Young leaf2 | 46,144,530 | 44,012,286 (95.38%) | 1,154,467 (2.5%) | 42,857,819 (92.88%) |
| Young leaf3 | 54,696,468 | 52,192,926 (95.42%) | 1,388,869 (2.54%) | 50,804,057 (92.88%) |
| Mature leaf1 | 43,638,108 | 41,654,536 (95.45%) | 1,081,191 (2.48%) | 40,573,345 (92.98%) |
| Mature leaf2 | 47,662,858 | 45,493,900 (95.45%) | 1,170,234 (2.46%) | 44,323,666 (92.99%) |
| Mature leaf3 | 45,185,150 | 43,078,939 (95.34%) | 1,078,796 (2.39%) | 42,000,143 (92.95%) |
| Stem xylem1 | 43,241,248 | 41,116,730 (95.09%) | 869,194 (2.01%) | 40,247,536 (93.08%) |
| Stem xylem2 | 45,714,492 | 43,573,131 (95.32%) | 917,872 (2.01%) | 42,655,259 (93.31%) |
| Stem xylem3 | 47,510,830 | 45,269,405 (95.28%) | 939,907 (1.98%) | 44,329,498 (93.3%) |
| Stem phloem1 | 45,306,012 | 43,092,457 (95.11%) | 1,001,985 (2.21%) | 42,090,472 (92.9%) |
| Stem phloem2 | 45,437,264 | 43,250,529 (95.19%) | 1,008,244 (2.22%) | 42,242,285 (92.97%) |
| Stem phloem3 | 47,900,044 | 45,589,087 (95.18%) | 1,104,828 (2.31%) | 44,484,259 (92.87%) |
| Principal root1 | 44,695,002 | 42,445,328 (94.97%) | 1,053,813 (2.36%) | 41,391,515(92.61%) |
| Principal root2 | 46,191,980 | 43,903,235 (95.05%) | 1,087,491 (2.35%) | 42,815,744 (92.69%) |
| Principal root3 | 44,956,244 | 42,613,889 (94.79%) | 1,139,361 (2.53%) | 41,474,528 (92.26%) |
| Secondary lateral root1 | 47,828,434 | 45,413,553 (94.95%) | 1,115,866 (2.33%) | 44,297,687(92.62%) |
| Secondary lateral root2 | 42,857,354 | 40,651,798 (94.85%) | 989,803 (2.31%) | 39,661,995 (92.54%) |
| Secondary lateral root3 | 43,332,426 | 40,969,891 (94.55%) | 1,012,416 (2.34%) | 39,957,475 (92.21%) |
| Fibrous root1 | 46,574,578 | 44,048,330 (94.58%) | 1,101,682 (2.37%) | 42,946,648 (92.21%) |
| Fibrous root2 | 44,387,462 | 41,773,074 (94.11%) | 999,976 (2.25%) | 40,773,098 (91.86%) |
| Fibrous root3 | 49,119,890 | 46,569,563 (94.81%) | 1,122,866 (2.29%) | 45,446,697 (92.52%) |

**Table S3 Distribution of reads in different regions.**

| Sample | CDS | Intergenic | Introns | 3'UTR | 5'UTR |
| --- | --- | --- | --- | --- | --- |
| Young leaf1 | 52,145,794 (82.0%) | 2,105,706 (3.31%) | 4,061,748 (6.39%) | 3,214,075 (5.05%) | 2,068,023 (3.25%) |
| Young leaf2 | 52,377,722 (82.01%) | 2,141,844 (3.35%) | 4,020,182 (6.29%) | 3,258,914 (5.1%) | 2,067,536 (3.24%) |
| Young leaf3 | 62,488,842 (82.35%) | 2,540,174 (3.35%) | 4,682,753 (6.17%) | 3,621,397 (4.77%) | 2,546,981 (3.36%) |
| Mature leaf1 | 50,056,309 (82.09%) | 1,877,305 (3.08%) | 4,129,537 (6.77%) | 2,792,541 (4.58%) | 2,121,889 (3.48%) |
| Mature leaf2 | 54,892,931 (82.2%) | 2,152,826 (3.22%) | 4,329,062 (6.48%) | 3,110,964 (4.66%) | 2,296,263 (3.44%) |
| Mature leaf3 | 51,312,989 (81.91%) | 2,071,689 (3.31%) | 4,102,043 (6.55%) | 2,964,822 (4.73%) | 2,192,070 (3.5%) |
| Stem xylem1 | 50,020,162 (83.35%) | 1,600,267 (2.67%) | 3,434,236 (5.72%) | 2,634,684 (4.39%) | 2,322,601 (3.87%) |
| Stem xylem2 | 52,895,366 (83.42%) | 1,658,078 (2.61%) | 3,625,877 (5.72%) | 2,783,535 (4.39%) | 2,447,200 (3.86%) |
| Stem xylem3 | 55,121,491 (83.82%) | 1,709,000 (2.6%) | 3,614,820 (5.5%) | 2,801,691 (4.26%) | 2,515,398 (3.82%) |
| Stem phloem1 | 50,463,368 (81.58%) | 1,790,936 (2.9%) | 4,126,822 (6.67%) | 3,322,886 (5.37%) | 2,154,533 (3.48%) |
| Stem phloem2 | 50,562,399 (81.55%) | 1,785,521 (2.88%) | 4,186,161 (6.75%) | 3,313,648 (5.34%) | 2,155,409 (3.48%) |
| Stem phloem3 | 53,649,130 (82.12%) | 1,880,874 (2.88%) | 4,148,752 (6.35%) | 3,507,401 (5.37%) | 2,146,440 (3.29%) |
| Principal root1 | 49,719,129 (82.03%) | 1,686,572 (2.78%) | 4,020,905 (6.63%) | 2,941,124 (4.85%) | 2,246,814 (3.71%) |
| Principal root2 | 51,194,894 (81.77%) | 1,737,887 (2.78%) | 4,253,081 (6.79%) | 3,139,299 (5.01%) | 2,284,358 (3.65%) |
| Principal root3 | 50,431,570 (82.53%) | 1,638,920 (2.68%) | 3,971,649 (6.5%) | 2,782,007 (4.55%) | 2,280,003 (3.73%) |
| Secondary lateral root1 | 50,917,658 (80.83%) | 1,811,341 (2.88%) | 4,909,702 (7.79%) | 3,313,649 (5.26%) | 2,040,918 (3.24%) |
| Secondary lateral root2 | 45,557,734 (80.73%) | 1,643,517 (2.91%) | 4,337,627 (7.69%) | 3,023,887 (5.36%) | 1,868,229 (3.31%) |
| Secondary lateral root3 | 45,834,299 (80.86%) | 1,658,612 (2.93%) | 4,328,505 (7.64%) | 2,859,324 (5.04%) | 2,004,987 (3.54%) |
| Fibrous root1 | 50,079,300 (81.58%) | 1,728,877 (2.82%) | 4,410,739 (7.19%) | 3,200,324 (5.21%) | 1,965,128 (3.2%) |
| Fibrous root2 | 47,281,611 (81.47%) | 1,628,653 (2.81%) | 4,257,495 (7.34%) | 2,968,224 (5.11%) | 1,899,852 (3.27%) |
| Fibrous root3 | 52,860,763 (81.42%) | 1,818,443 (2.8%) | 4,808,114 (7.41%) | 3,210,903 (4.95%) | 2,225,131 (3.43%) |

**Table S4 Summary of abiotic elicitor-treated transcriptome and reference genome alignment results.**

| Sample | Total reads | Total mapped | Multiple mapped | Uniquely mapped |
| --- | --- | --- | --- | --- |
| CG_1 | 46,788,402 | 44,396,874 (94.89%) | 1,124,800 (2.4%) | 43,272,074 (92.48%) |
| CG_2 | 52,545,082 | 49,947,245 (95.06%) | 1,184,129 (2.25%) | 48,763,116 (92.8%) |
| CG_3 | 51,574,944 | 49,579,241 (96.13%) | 1,221,787 (2.37%) | 48,357,454 (93.76%) |
| AgNO_3__1 | 60,134,814 | 57,478,945 (95.58%) | 1,673,436 (2.78%) | 55,805,509 (92.8%) |
| AgNO_3__2 | 54,757,794 | 52,536,931 (95.94%) | 1,414,954 (2.58%) | 51,121,977 (93.36%) |
| AgNO_3__3 | 53,911,594 | 51,688,549 (95.88%) | 1,564,561 (2.9%) | 50,123,988 (92.97%) |
| PEG_1 | 49,850,560 | 47,564,578 (95.41%) | 1,088,951 (2.18%) | 46,475,627 (93.23%) |
| PEG_2 | 49,479,170 | 46,631,571 (94.24%) | 1,107,844 (2.24%) | 45,523,727 (92.01%) |
| PEG_3 | 56,039,550 | 53,326,666 (95.16%) | 1,596,660 (2.85%) | 51,730,006 (92.31%) |
| MeJA_1 | 48,715,302 | 46,345,824 (95.14%) | 1,056,676 (2.17%) | 45,289,148 (92.97%) |
| MeJA_2 | 55,693,218 | 53,219,060 (95.56%) | 1,333,745 (2.39%) | 51,885,315 (93.16%) |
| MeJA_3 | 52,837,940 | 50,038,718 (94.7%) | 1,196,905 (2.27%) | 48,841,813 (92.44%) |

**Table S5 Distribution of reads in different regions in abiotic elicitor-treated transcriptome.**

| Sample | CDS | Intergenic | Introns | 3'UTR | 5'UTR |
| --- | --- | --- | --- | --- | --- |
| CG_1 | 48,227,231 (78.61%) | 2,354,798 (3.84%) | 4,627,350 (7.54%) | 4,034,820 (6.58%) | 2,107,688 (3.44%) |
| CG_2 | 54,647,767 (78.87%) | 2,719,751 (3.93%) | 5,178,049 (7.47%) | 4,470,771 (6.45%) | 2,274,655 (3.28%) |
| CG_3 | 55,725,983 (79.66%) | 2,923,212 (4.18%) | 4,520,962 (6.46%) | 4,569,043 (6.53%) | 2,218,449 (3.17%) |
| AgNO_3__1 | 61,591,402 (78.42%) | 3,236,192 (4.12%) | 5,594,859 (7.12%) | 5,563,701 (7.08%) | 2,553,187 (3.25%) |
| AgNO_3__2 | 56,931,020 (78.43%) | 2,831,046 (3.9%) | 5,536,261 (7.63%) | 4,803,296 (6.62%) | 2,483,404 (3.42%) |
| AgNO_3__3 | 55,727,710 (78.45%) | 2,780,494 (3.91%) | 5,240,720 (7.38%) | 4,975,044 (7.0%) | 2,311,730 (3.25%) |
| PEG_1 | 51,929,973 (79.44%) | 2,193,376 (3.36%) | 4,664,743 (7.14%) | 4,450,923 (6.81%) | 2,128,867 (3.26%) |
| PEG_2 | 48,958,560 (77.92%) | 2,308,522 (3.67%) | 4,823,634 (7.68%) | 5,022,595 (7.99%) | 1,721,404 (2.74%) |
| PEG_3 | 56,745,756 (78.21%) | 2,758,997 (3.8%) | 5,480,018 (7.55%) | 5,234,331 (7.21%) | 2,337,327 (3.22%) |
| MeJA_1 | 48,624,596 (79.4%) | 1,994,729 (3.26%) | 4,980,565 (8.13%) | 3,672,548 (6.0%) | 1,969,223 (3.22%) |
| MeJA_2 | 56,845,516 (79.1%) | 2,343,601 (3.26%) | 5,696,166 (7.93%) | 4,733,243 (6.59%) | 2,243,072 (3.12%) |
| MeJA_3 | 54,074,927 (79.27%) | 2,261,598 (3.32%) | 5,138,190 (7.53%) | 4,481,865 (6.57%) | 2,263,356 (3.32%) |

**Table S6 Summary of reported gene clusters for camptothecin biosynthesis.**

| Gene ID | Annotation | Genomic location | Strand |
| --- | --- | --- | --- |
| *CacGene09399* | Isopentenyl diphosphate isomerase | Lachesis_group 16: 12,440,392-12,448,294 | Rev |
| *CacGene09411* | 7-Deoxyloganic acid hydroxylase/Secologanic acid synthase | Lachesis_group 16: 12,309,990-12,314,190 | Rev |
| *CacGene09419* | *O-*Methyltransferase | Lachesis_group 16: 12,209,416-12,214,040 | Fwd |
| *CacGene09425* | Alcohol dehydrogenase | Lachesis_group 16: 12,095,205-12,096,430 | Fwd |
| *CacGene09426* | 10-Hydroxygeraniol oxidoreductase | Lachesis_group 16: 12,091,007-12,094,198 | Fwd |
| *CacGene09428* | Alcohol dehydrogenase | Lachesis_group 16: 12,067,102-12,071,061 | Fwd |
| *CacGene09429* | 10-Hydroxygeraniol oxidoreductase | Lachesis_group 16: 12,067,936-12,069,486 | Fwd |
| *CacGene09430* | 10-Hydroxygeraniol oxidoreductase | Lachesis_group 16: 12,067,700-12,065,124 | Fwd |

**Table S7 Summary of gene clusters for camptothecin biosynthesis.**

| Gene ID | Annotation | Genomic location | Strand |
| --- | --- | --- | --- |
| *CacGene23490* | *O*-Methyltransferase | Lachesis_group 8: 11,266,227-11,269,715 | Fwd |
| *CacGene23491* | *O-*Methyltransferase | Lachesis_group 8: 11,306,232-11,324,284 | Fwd |
| *CacGene23492* | *O*-Methyltransferase | Lachesis_group 8: 11,322,971-11,335,912 | Fwd |
| *CacGene23494* | 10-Hydroxycamptothecin *O*-methyltransferase | Lachesis_group 8: 11,346,267-11,347,804 | Fwd |
| *CacGene23496* | 1-Deoxy-D-xylulose 5-phosphate synthase | Lachesis_group 8: 11,369,075-11,372,589 | Rev |

**Table S8 Summary of the predicted properties of OMTs.**

| NO. | Gene ID | Amino acid length (aa) | pI | MW |
| --- | --- | --- | --- | --- |
| 1 | *CacGene01857* | 332 | 7.3 | 36,656.81 |
| 2 | *CacGene02488* | 348 | 5.19 | 38,313.49 |
| 3 | *CacGene04089* | 231 | 5.26 | 26,074.84 |
| 4 | *CacGene05669* | 295 | 8.57 | 31,955.21 |
| 5 | *CacGene05741* | 368 | 5.15 | 41,695.76 |
| 6 | *CacGene05742* | 368 | 5.52 | 42,001.85 |
| 7 | *CacGene05950* | 358 | 5.30 | 39,440.61 |
| 8 | *CacGene08300* | 372 | 5.23 | 41,758.30 |
| 9 | *CacGene08456* | 358 | 5.43 | 39,068.20 |
| 10 | *CacGene08457* | 337 | 5.60 | 36,762.63 |
| 11 | *CacGene09500* | 364 | 5.43 | 40,001.34 |
| 12 | *CacGene09630* | 238 | 5.66 | 26,664.99 |
| 13 | *CacGene13882* | 358 | 5.46 | 39,499.49 |
| 14 | *CacGene14160* | 468 | 5.77 | 52,501.27 |
| 15 | *CacGene16005* | 235 | 5.66 | 26,542.73 |
| 16 | *CacGene16008* | 363 | 5.18 | 40,720.24 |
| 17 | *CacGene16009* | 372 | 5.27 | 41,662.19 |
| 18 | *CacGene17405* | 298 | 5.37 | 33,587.51 |
| 19 | *CacGene17407* | 359 | 4.82 | 39,275.48 |
| 20 | *CacGene19078* | 354 | 5.54 | 40,488.11 |
| 21 | *CacGene19151* | 289 | 7.67 | 31,931.95 |
| 22 | *CacGene21154* | 357 | 5.41 | 39,394.35 |
| 23 | *CacGene21628* | 304 | 8.86 | 33,944.33 |
| 24 | *CacGene21790* | 247 | 5.71 | 27,832.93 |
| 25 | *CacGene22519* | 362 | 5.31 | 40,802.06 |
| 26 | *CacGene22567* | 385 | 5.35 | 42,470.07 |
| 27 | *CacGene22716* | 364 | 5.64 | 40,027.40 |
| 28 | *CacGene22777* | 361 | 5.42 | 39,602.47 |
| 29 | *CacGene22778* | 361 | 5.36 | 39,580.53 |
| 30 | *CacGene23490* | 330 | 5.41 | 37,319.74 |
| 31 | *CacGene23491* | 355 | 5.37 | 39,809.82 |
| 32 | *CacGene23492* | 355 | 5.43 | 40,117.18 |
| 33 | *CacGene23494* | 355 | 5.22 | 39,970.03 |
| 34 | *CacGene23801* | 360 | 6.14 | 40,710.60 |
| 35 | *CacGene23803* | 366 | 6.43 | 40,988.39 |
| 36 | *CacGene24388* | 379 | 8.16 | 42,598.78 |
| 37 | *CacGene26256* | 283 | 8.25 | 30,833.46 |
| 38 | *CacGene26520* | 354 | 5.56 | 39,023.29 |
| 39 | *CacGene26521* | 263 | 5.90 | 28,728.15 |
| 40 | *CacGene27071* | 316 | 5.40 | 35,928.58 |

**Table S9 The Ka, Ks, and Ka/Ks values of *CaOMT* genes pairs.**

| Gene pair | Ka | Ks | Ka/Ks |
| --- | --- | --- | --- |
| *CaOMT1*-*CaOMT2* | 0.038 | 0.049 | 0.762 |
| *CaOMT1*-*CaOMT3* | 0.026 | 0.050 | 0.522 |
| *CaOMT1*-*CaOMT4* | 0.043 | 0.062 | 0.693 |
| *CaOMT2*-*CaOMT3* | 0.033 | 0.041 | 0.805 |
| *CaOMT2*-*CaOMT4* | 0.016 | 0.038 | 0.412 |
| *CaOMT3*-*CaOMT4* | 0.033 | 0.056 | 0.592 |

**Table S10 Summary of amino acid sequences used in the phylogenetic tree.**

| **Abbreviation** | **Plant species** | **Protein function** | **Accession no.** |
| --- | --- | --- | --- |
| AtFlav3'-CaffOMT | *Arabidopsis thaliana* | O-methyltransferase | AAM10127 |
| AtgTMT | *A. thaliana* | Gamma-tocopherol methyltransferase | AAD02882 |
| AtIAMT | *A. thaliana* | Indole-3-acetate O-methyltransferase | Q9FLN8 |
| AtJMT | *A. thaliana* | Jasmonate O-methyltransferase | Q9AR07 |
| AtSMT | *A. thaliana* | Sterol methyltransferase | NP_001078579 |
| CaOMT1 | *Camptotheca acuminata* | 10-Hydroxycamptothecin O-methyltransferase | AWH62806 |
| CaFOMT1 | *C. acuminata* | Flavonol O-methyltransferase | / |
| CaFOMT2 | *C. acuminata* | Flavonol O-methyltransferase | / |
| CamOMT | *Chrysosplenium americanum* | 3' Flavonoid O-methyltransferase | AAA80579 |
| CaOMT2 | *C. acuminata* | O-methyltransferase |  |
| CaOMT3 | *C. acuminata* | O-methyltransferase |  |
| CaOMT4 | *C. acuminata* | O-methyltransferase |  |
| CarMXNMT | *Coffea arabica* | 7-Methylxanthine N-methyltransferase | BAB39216 |
| CbSAMT | *Clarkia breweri* | S-Adenosyl-L-methionine: salicylic acid carboxyl methyltransferase | AF133053 |
| CdFlav3/3'/5/6/7OMT | *Citrus depressa* | O-methyltransferase | BAU51794 |
| CjCNMT | *Coptis japonica* | Coclaurine *N*-methyltransferase | BAB71802 |
| CjNM4OMT | *C. japonica* | 3'-Hydroxy-N-methyl-(S)-coclaurine 4'-O-methyltransferase | Q9LEL5 |
| Cr16OMT | *Catharanthus roseus* | 16-Hydroxytabersonine O-methyltransferase | ABR20103 |
| CrCaffAcOMT | *C. roseus* | Caffeic acid O-methyltransferase | AAK20170 |
| CrFlav3'/5'OMT | *C. roseus* | O-methyltransferase | AAM97498 |
| CrFlav4'OMT | *C. roseus* | Flavonoid 4'-O-methyltransferase | AAR02419 |
| CrLAMT | *C. roseus* | Loganic acid O-methyltransferase | AGX93063 |
| CrNMT | *C. roseus* | 16-Hydroxy-2,3-dihydro-3-hydroxytabersonine *N*-methyltransferase | AHH33092 |
| CrOMT | *C. reticulata* | Caffeic acid O-methyltransferase | ADK97702 |
| CsCafSyn | *Camellia sinensis* | Caffeine synthase | BAB12278 |
| CuOMT | *C. sinensis* | Caffeine synthase | BBU25484 |
| HvFlavOMT | Hordeum vulgare subsp. vulgare | Flavonoid 7-O-methyltransferase | CAA54616 |
| MpOMT3 | Mentha x piperita | Flavonoid 3'-O-methyltransferase | AAR09601 |
| Ms7IOMT | *Medicago sativa* | 7-O-Methyltransferase | AAC49927 |
| MsCaffAcOMT | *M. sativa* | Caffeic acid 3-O-methyltransferase | AAB46623 |
| MsCaffCoA-3OMT | *M. sativa* | trans-Caffeoyl-CoA 3-O-methyltransferase | AAC28973 |
| MtIsoflav-6aOHmaack3OMT | *M. truncatula* | SAM Dependent isoflavanone 4'-O-methyltransferase | AAY18581 |
| NpN4'OMT | Narcissus pseudonarcissus | Norbelladine 4'-O-methyltransferase | AIL54541 |
| NtCCoAOMT | *Nicotiana tabacum* | Caffeoyl-coenzyme A O-methyltransferase | AAC49913 |
| NtSMT | *N. tabacum* | Cycloartenol-C-24-methyltransferase | NP_001312832 |
| OsOMT | Oryza sativa Japonica Group | O-methyltransferase | ABB90678 |
| CiOMT | *Carapichea ipecacuanha* | O-methyltransferase | BAI79243 |
| Ps4OMT | *Papaver somniferum* | 3'-Hydroxy-N-methyl-(S)-coclaurine 4'-O-methyltransferase | Q7XB10 |
| Ps6OMT | *P. somniferum* | (R,S)-Norcoclaurine 6-O-methyltransferase | AAQ01669 |
| PsCNMT | *P. somniferum* | Coclaurine N-methyltransferase | AAP45316 |
| PsN7OMT | *P. somniferum* | Norreticuline-7-O-methyltransferase | ACN88562 |
| PsTNMT | *P. somniferum* | (S)-Tetrahydroprotoberberine-cis-N-methyltransferase | AAY79177 |
| PtCCoAOMT | *Populus tremuloides* | Caffeoyl-CoA 3-O-methyltransferase | AAA80651 |
| RhOrcOMT | Rosa hybrid cultivar | Orcinol O-methyltransferase | CAH05087 |
| RsPiNMT | *Rauvolfia serpentina* | Tocopherol-like methyltransferase | AHH02780 |
| ShFlav3'/5'OMT | *Solanum habrochaites* | Myricetin *O*-methyltransferase | ADZ76433 |
| ShFlav3OMT | *S. habrochaites* | Flavonol 3-O-methyltransferase | AGK26768 |
| ShFlav7/4'OMT | *S. habrochaites* | Myricetin O-methyltransferase | ADZ76434 |
| ShOMT1 | *S. habrochaites* | Myricetin O-methyltransferase | ADZ76433 |
| ShOMT3 | *S. habrochaites* | Flavonol 3-*O*-methyltransferase | AGK26768 |
| TtOMT | *Thalictrum tuberosum* | Catechol O-methyltransferase | AAD29841 |
| TaOMT1 | *Triticum aestivum* | Flavone *O*-methyltransferase | Q84N28 |
| TaOMT2 | *T. aestivum* | Flavone *O*-methyltransferase | ABB03907 |
| TfCNMT | T. flavum subsp. glaucum | (S)-Coclaurine N-methyltransferase | AAU20766 |
| TfNc6OMT | T. flavum subsp. glaucum | (S)-Norcoclaurine 6-O-methyltransferase | AAU20765 |
| TtCaffAc-IsoquiAlkOMT | *T. tuberosum* | Catechol O-methyltransferase | AAD29841 |
| VmPiNMT | *Vinca minor* | Tocopherol-like methyltransferase | AHH02782 |
| VpOMT4 | *Vanilla planifolia* | O-methyltransferase | ADZ76153 |
| VvCCoAOMT | *Vitis vinifera* | Caffeoyl-CoA O-methyltransferase | CAA90969 |
| VvResOMT | *V. vinifera* | Resveratrol O-methyltransferase | CAQ76879 |

**Table S11 Summary of primers in this study.**

| **Primer** | **Sequence (5’ to 3’)** | **Purpose** |
| --- | --- | --- |
| *CaOMT1/CaOMT2/CaOMT3-F* | ATGGCTAATGATGAGAGATATAC | Gene cloning |
| *CaOMT1/CaOMT2/CaOMT3-R* | TTAAGGATAAACCTCAATGAGAG | Gene cloning |
| *pCold-TF-CaOMT4-BamHI-F* | ctcggtaccctcgagggatccATGGCTAATGAGGAGAGATATAC | Gene cloning |
| *pCold-TF-CaOMT1-3 -BamHI-F* | ctcggtaccctcgagggatccATGGCTAATGATGAGAGATATAC | Gene cloning |
| *pCold-TF-CaOMT1-4-EcoRI-R* | caggtcgacaagcttgaattcTTAAGGATAAACCTCAATGAGAG | Gene cloning |
| *CaOMT3-F116A-F* | TTGAATGCAAGGGCAGCCTTGCTTGCAGTTATCAATCC | Site-directed mutagenesis |
| *CaOMT3-F116A-R* | GGATTGATAACTGCAAGCAAGGCTGCCCTTGCATTCAA | Site-directed mutagenesis |
| *CaOMT3-A119V-F* | AGGGCATTCTTGCTTGTAGTTATCAATCCAATCATGAC | Site-directed mutagenesis |
| *CaOMT3-A119V-R* | GTCATGATTGGATTGATAACTACAAGCAAGAATGCCCT | Site-directed mutagenesis |
| *CaOMT3-V120A-F* | GCATTCTTGCTTGCAGCTATCAATCCAATCATGACAAA | Site-directed mutagenesis |
| *CaOMT3-V120A-R* | TTTGTCATGATTGGATTGATAGCTGCAAGCAAGAATGC | Site-directed mutagenesis |
| *CaOMT3-M125A-F* | TTATCAATCCAATCGCGACAAAACCATGGCATTTCTTGAGT | Site-directed mutagenesis |
| *CaOMT3-M125A-R* | ACTCAAGAAATGCCATGGTTTTGTCGCGATTGGATTGATAA | Site-directed mutagenesis |
| *CaOMT3-F152A-F* | GCACATGGAAGAAGTGCTTGGGATTATCTTGCTCAAGA | Site-directed mutagenesis |
| *CaOMT3-F152A-R* | TCTTGAGCAAGATAATCCCAAGCACTTCTTCCATGTGC | Site-directed mutagenesis |
| *CaOMT3-L156A-F* | GTTTTTGGGATTATGCTGCTCAAGAGCCAAGACTCGGAAAT | Site-directed mutagenesis |
| *CaOMT3-L156A-R* | ATTTCCGAGTCTTGGCTCTTGAGCAGCATAATCCCAAAAAC | Site-directed mutagenesis |
| *CaOMT3-F166A-F* | AGACTCGGAAATTTTGCTGACGAAGCCATGGCAAGTGA | Site-directed mutagenesis |
| *CaOMT3-F166A-R* | TCACTTGCCATGGCTTCGTCAGCAAAATTTCCGAGTCT | Site-directed mutagenesis |
| *CaOMT3-W257A-F* | GCAATTTTACTCAAGGCGATTCTGCACAATTGGTCTGA | Site-directed mutagenesis |
| *CaOMT3-W257A-R* | TCAGACCAATTGTGCAGAATCGCCTTGAGTAAAATTGC | Site-directed mutagenesis |
| *CaOMT3-H260A-F* | CTCAAGTGGATTCTGGCCAATTGGTCTGAGGAAGAATG | Site-directed mutagenesis |
| *CaOMT3-H260A-R* | CATTCTTCCTCAGACCAATTGGCCAGAATCCACTTGAG | Site-directed mutagenesis |
| *CaOMT3-N261A-F* | AAGTGGATTCTGCACGCTTGGTCTGAGGAAGAATGTGT | Site-directed mutagenesis |
| *CaOMT3-N261A-R* | ACACATTCTTCCTCAGACCAAGCGTGCAGAATCCACTT | Site-directed mutagenesis |
| *CaOMT3-S309A-F* | AGGCACAGCTCTCTGCTGACATGACGATGATGGTTCTATTC | Site-directed mutagenesis |
| *CaOMT3-S309A-R* | GAATAGAACCATCATCGTCATGTCAGCAGAGAGCTGTGCCT | Site-directed mutagenesis |
| *CaOMT3-T312A-F* | TCTCTTCTGACATGGCGATGATGGTTCTATTCAGAGGTCAA | Site-directed mutagenesis |
| *CaOMT3-T312A-R* | TTGACCTCTGAATAGAACCATCATCGCCATGTCAGAAGAGA | Site-directed mutagenesis |
| *CaOMT3-M313A-F* | TCTTCTGACATGACGGCGATGGTTCTATTCAGAGGTCA | Site-directed mutagenesis |
| *CaOMT3-M313A-R* | TGACCTCTGAATAGAACCATCGCCGTCATGTCAGAAGA | Site-directed mutagenesis |
| *CaOMT3-F317A-F* | CGATGATGGTTCTAGCCAGAGGTCAAGAGAGAAGTGAGAAA | Site-directed mutagenesis |
| *CaOMT3-F317A-R* | TTTCTCACTTCTCTCTTGACCTCTGGCTAGAACCATCATCG | Site-directed mutagenesis |
| *CaOMT3-M125L-F* | GTTATCAATCCAATCCTGACAAAACCATGGCATTTCTT | Site-directed mutagenesis |
| *CaOMT3-M125L-R* | AAGAAATGCCATGGTTTTGTCAGGATTGGATTGATAAC | Site-directed mutagenesis |
| *CaOMT3-L156G-F* | AGTTTTTGGGATTATGGTGCTCAAGAGCCAAGACTCGG | Site-directed mutagenesis |
| *CaOMT3-L156G-R* | CCGAGTCTTGGCTCTTGAGCACCATAATCCCAAAAACT | Site-directed mutagenesis |
| *CaOMT3-N261D-F* | AAGTGGATTCTGCACGATTGGTCTGAGGAAGAATGTGT | Site-directed mutagenesis |
| *CaOMT3-N261D-R* | ACACATTCTTCCTCAGACCAATCGTGCAGAATCCACTT | Site-directed mutagenesis |
| *CaOMT3-S309F-F* | GAGGCACAGCTCTCTTTTGACATGACGATGATGGTTCT | Site-directed mutagenesis |
| *CaOMT3-S309F-R* | AGAACCATCATCGTCATGTCAAAAGAGAGCTGTGCCTC | Site-directed mutagenesis |
| *CaOMT3-T312L-F* | CTCTCTTCTGACATGCTGATGATGGTTCTATTCAGAGG | Site-directed mutagenesis |
| *CaOMT3-T312L-R* | CCTCTGAATAGAACCATCATCAGCATGTCAGAAGAGAG | Site-directed mutagenesis |
| *CaOMT3-F317L-F* | ACGATGATGGTTCTACTCAGAGGTCAAGAGAGAAGTGA | Site-directed mutagenesis |
| *CaOMT3-F317L-R* | TCACTTCTCTCTTGACCTCTGAGTAGAACCATCATCGT | Site-directed mutagenesis |
